# Cerebral organoids with chromosome 21 trisomy secrete Alzheimer’s disease-related soluble aggregates detectable by single-molecule-fluorescence and super-resolution microscopy

**DOI:** 10.1038/s41380-023-02333-3

**Published:** 2023-12-15

**Authors:** Emre Fertan, Dorothea Böken, Aoife Murray, John S. H. Danial, Jeff Y. L. Lam, Yunzhao Wu, Pollyanna A. Goh, Ivan Alić, Matthew R. Cheetham, Evgeniia Lobanova, Yu P. Zhang, Dean Nižetić, David Klenerman

**Affiliations:** 1 Yusuf Hamied Department of Chemistry, University of Cambridge, Cambridge, CB2 1EW UK; 2 UK Dementia Research Institute at University of Cambridge, Cambridge, CB2 0AH UK; 3 The Blizard Institute, Barts & The London School of Medicine, Queen Mary University of London, London, E1 2AT UK; 4 Lee Kong Chian School of Medicine, Nanyang Technological University, Singapore, Singapore; 5 Department of Anatomy, Histology and Embryology, Faculty of Veterinary Medicine, University of Zagreb, Zagreb, Croatia

**Keywords:** Diseases, Molecular biology, Stem cells

## Abstract

Understanding the role of small, soluble aggregates of beta-amyloid (Aβ) and tau in Alzheimer’s disease (AD) is of great importance for the rational design of preventative therapies. Here we report a set of methods for the detection, quantification, and characterisation of soluble aggregates in conditioned media of cerebral organoids derived from human iPSCs with trisomy 21, thus containing an extra copy of the amyloid precursor protein (APP) gene. We detected soluble beta-amyloid (Aβ) and tau aggregates secreted by cerebral organoids from both control and the isogenic trisomy 21 (T21) genotype. We developed a novel method to normalise measurements to the number of live neurons within organoid-conditioned media based on glucose consumption. Thus normalised, T21 organoids produced 2.5-fold more Aβ aggregates with a higher proportion of larger (300–2000 nm^2^) and more fibrillary-shaped aggregates than controls, along with 1.3-fold more soluble phosphorylated tau (pTau) aggregates, increased inflammasome ASC-specks, and a higher level of oxidative stress inducing thioredoxin-interacting protein (TXNIP). Importantly, all this was detectable prior to the appearance of histological amyloid plaques or intraneuronal tau-pathology in organoid slices, demonstrating the feasibility to model the initial pathogenic mechanisms for AD in-vitro using cells from live genetically pre-disposed donors before the onset of clinical disease. Then, using different iPSC clones generated from the same donor at different times in two independent experiments, we tested the reproducibility of findings in organoids. While there were differences in rates of disease progression between the experiments, the disease mechanisms were conserved. Overall, our results show that it is possible to non-invasively follow the development of pathology in organoid models of AD over time, by monitoring changes in the aggregates and proteins in the conditioned media, and open possibilities to study the time-course of the key pathogenic processes taking place.

## Introduction

Alzheimer’s disease (AD) is a progressive, neurodegenerative disorder, leading to gliosis, synaptic loss, and cerebral atrophy, accompanied by physiological, cognitive, and behavioural deficits [1–5]. With a prevalence rate exceeding 5% of the population in Europe [6], AD is the most common cause of dementia. While the exact cause(s) and progression mechanisms of AD remain elusive, the accumulation of certain proteins and peptides have been studied and hypothesised to contribute to the pathogenesis.

The build-up of intercellular ‘plaques’ in AD was first documented by Alois Alzheimer [7] and later the composition of these plaques was identified to be mainly beta-amyloid (Aβ) [8], which gave rise to the amyloid cascade hypothesis [9–11], postulating the accumulation of Aβ as the primary cause of AD. Over the years, the cascade hypothesis has been heavily criticised for a number of reasons, mainly due to the observation of Aβ plaques in the brains of non-demented individuals [12, 13], initial failure of drug trials targeting Aβ [14–16], and the identification of other disease mechanisms such as neuroinflammation, oxidative stress, and pathological accumulation of microtubule-associated protein tau [17, 18]. However, findings from more recent studies identifying the small (oligomeric) soluble species of Aβ as the more toxic ones instead of the plaques [19, 20], and linking these aggregates to other disease mechanisms such as inflammation, altered gene expression, and tau pathology [21–24] updated and strengthened the cascade hypothesis [25]. The main remaining challenge is detecting and characterising these small aggregates in physiologically relevant systems -due to their size, low abundance, and heterogeneity.

One of the major reinforcers of the amyloid cascade hypothesis is the link between Down’s syndrome (DS) and AD. As the most common survivable chromosomal disorder in humans [26–29] cognitive and behavioural phenotype of DS include intellectual disability with difficulty in verbal communication, delayed and reduced motor development, and an increased predisposition to psychiatric conditions [30–33]. Unlike many neurological disorders, the cause of DS is well understood and genetically defined as the triplication of the twenty-first chromosome (trisomy 21, (T21)). This can occur in multiple ways; most often as a complete trisomy in all the cells, or (rarer) as mosaic T21, affecting some of the somatic cells [34, 35].

During the first half of the 20th century, the average life expectancy for children with DS was 9 years [36], however, due to the advancements in neurobiology and healthcare in general, the average lifespan of individuals with DS has been increased to more than 50 years [37], with an increasing number living into their 70s. This increased longevity, however leads to a significantly increased risk of AD in people with DS, compared to euploid individuals [38, 39]. This is caused by the over-expression of amyloid precursor protein gene (*APP*), which is located on chromosome 21 (chr21) and is processed to produce Aβ. At least two cases have been reported on persons born with a trisomy of only part of chr21 that did not include *APP*, with DS but lacking Aβ pathology in their brains and did not develop dementia [40, 41] suggesting it is the trisomy of *APP* that confers the high risk for AD in the DS population. Conversely, if the section carrying the *APP* gene is duplicated and not the rest of chr21, individuals (familial condition called DupAPP) do not show signs of DS but do develop dementia with 100% penetrance before the age of 60 [42].

Recently we have generated cerebral organoids using iPSCs derived from individuals with DS [43] (Supplementary Fig. 1). AD-like pathology including extracellular Aβ plaque-like deposits, hyperphosphorylated, pathologically conformed tau, and premature neuronal loss could be detected in 5 of 7 cell donors with DS by 100 days in-vitro (DIV). However, no Aβ or tau pathology could be detected by immunohistochemistry in two of the samples throughout the experiment [43] (Supplemental Fig. 2). As mentioned above, instead of the insoluble plaques, the smaller, soluble, Aβ and tau aggregates, often referred to as oligomers, may be more toxic species, involved in AD pathogenesis. Due to the lack of suitable methods, it has so far not been explored if these small soluble species are present in the cerebral organoid DS models. Here we first studied conditioned media samples collected longitudinally between DIV 84 and 150 from one of the cell lines that did not show Aβ plaque pathology. Using single-molecule imaging and detection methods, we investigated if these organoids released aggregates into the media, if these secreted aggregates differed quantitatively and morphologically between T21 and D21 (control) organoids, and if they correlated with other AD and DS pathologies such as inflammation and oxidative stress. Then, using independent iPSC clones from the same donor at different times and by different experimentators, we repeated the key measurements from the first study, in order to test the reproducibility of organoids, which is an area of debate in the field [44, 45].

During these experiments, we used the single-molecule imaging methods our group and others have developed for super-resolution imaging of soluble aggregates -that are smaller than 200 nm, the diffraction limit of light, thus invisible to most detection techniques- to measure their size and determine how they change during the progression of AD [46–50]. Previously, using an aptamer that binds beta-sheet aggregates of both Aβ and alpha-synuclein, we found similar number of aggregates present in the cerebrospinal fluid (CSF) of patients with AD and healthy controls. These aggregates were small, on average 40 nm in length, but ranged from 30 to 200 nm, with a higher proportion of larger aggregates in AD patients [50]. Mildly cognitively impaired (MCI) patient CSF caused increased inflammation which correlated with the formation of a higher proportion of larger aggregates than controls [50], agreeing with our study of synthetic Aβ aggregates where we found that the mechanism of toxicity changed as the size of the aggregates increased [51]. However, our methods have only been used on human CSF, serum and post-mortem brain samples, to date. In this work, we adapted these methods to detect, quantitate, and characterise the soluble aggregates formed in a DS live cultured cerebral organoid model of AD over time.

## Methods

### Development of the cerebral organoids and conditioned media collection

Disomic and trisomic isogenic human iPSCs NIZEDSM1iD21‐C3, C9 (D21-C3/9) and NIZEDSM1iT21‐C5, C6, C13 (T21-C5/6/13) [52] were cultured following standard feeder-free culture conditions using mTESR1 or E8 media on Geltrex coated plates (Supplementary Fig. 1). Cerebral organoids were generated following a published protocol [53] with minor modifications as described by Alić et al. [43] To form embryoid bodies (EBs), hiPSCs were washed once with PBS, then incubated with Gentle Cell Dissociation Solution (StemCell Technologies; Cat. 100-0485) for 4 min. This solution was aspirated and accutase (Thermo Scientific; Cat. A1110501) added and incubated for a further 4 min. mTESR1/E8 medium at double the volume of accutase was added and a single cell suspension generated by titrating. Cells were centrifuged to remove accutase and then resuspended in hESC medium supplemented with 4 ng/ml FGF2 and 50 μM ROCK inhibitor. In all, 9000 cells per well were used to form a single EB in a V-shaped ultra-low attachment 96-well plate (Corning). EBs were cultured in suspension for 6 days in hESC medium with low FGF. After 5–7 days, EBs were transferred into a 24-well ultra-low attachment plate for neural induction. Neural induction was achieved by culturing for further 5–7 days in DMEM-F12 supplemented with 1% of each: N2, GlutaMAX (Thermo Scientific; Cat. 35050061), and MEMNEAA (Thermo Scientific; Cat. 11140050), plus 1 μg/ml heparin. Induced EBs showing neuroectodermal “clearing” in brightfield microscopy were embedded in Matrigel droplets and transferred to 6 cm non-adherent dishes containing organoid differentiation medium-A, (for 4–5 days), followed by organoid differentiation medium+A. Organoid maturation was carried out with 12–16 organoids per 6 cm dish on an orbital shaker at 37 °C, 5% CO_2_. Organoids were fed and aliquots of conditioned media were collected from mature organoids at 3–4-day intervals, snap-frozen on dry ice, and stored at −80 °C.

### Glucose and LDH assays

Glucose and lactate dehydrogenase (LDH) concentrations in the conditioned media samples were measured using colorimetric assays (Glucose kit: abcam; Cat. ab65333, LDH kit: abcam; Cat. ab65393) following the assay instructions. In brief, for the glucose assay, 2 μL of media sample from each condition was mixed with 98 μL glucose assay buffer and loaded to a 96-well plate in duplicates at 50 μL per well, alongside the assay standards. 50 μL of reaction mix was added to each sample and standard well, gently shaken to mix, and incubated at 37 °C for 30 min, protected from light. Following the incubation, the plate was read at 570 nm.

For the LDH assay, 10 μL of media sample from each condition was loaded to the 96-well plate in duplicates and 100 μL LDH reaction mix (reconstituted in ddH2O) was added to each well. After gentle shaking to mix, and incubation at 23 °C for 30 min, the plate was read at 450 nm.

### SiMPull

Coverslips were prepared as described previously [48, 54, 55] and kept in vacuumed containers at −20 °C until used. At the beginning of each experiment, one vacuum box containing the coverslips was taken out of the freezer and placed inside a fume-hood for 60-min to equalise the temperature. Then, the coverslip was taken outside the vacuum box and placed inside a humid chamber. The coverslip was first passivated with neutravidin (0.2 mg/mL) diluted in 0.05% PBS-T(v/v) (tween20 Cat. P1379-25ML diluted in PBS) for 10-min. The neutravidin solution in each well was then removed, followed by washing with PBST (two cycles) and PBS with 1% Tween (one cycle)-hereon described as washing. For Aβ, hyper-phosphorylated tau, and ASC-speck capture, biotinylated 6E10 (BioLegend; Cat. 9340-02), AT8 (Invitrogen; Cat. MN1020B), and AL177 (AdipoGen; Cat. AG-25B-0006-C100) antibodies were used. The biotinylation of AL177 was done in house, as described in ‘Reproducibility of findings in DS organoids’. Capture antibodies were diluted to 10 nM in PBS containing 0.1 mg/ml bovine serum albumin (BSA; Thermo Scientific; Cat. 10829410) and incubated for 15 min. Then the wells were washed, and samples were incubated overnight at 4 °C. In order to minimise non-specific binding, a blocking step was performed for the tau and ASC-specks assays, with solution containing 3 mg/ml BSA incubated for 30 min, before the capture antibody and after the sample incubation, followed by washing. For detection, the Alexa-Fluor-647-labelled antibodies corresponding to the biotinylated capture antibody were diluted in BSA/PBS at 0.5 nM, 2 nM, and 1 nM concentrations and incubated for a duration of 45, 15, and 10 min for 6E10, AT8, and AL177, respectively followed by the washing step. Then 7 µL of PBS was added to each well and the coverslip was imaged using a purpose-built total internal reflection fluorescence (TIRF) microscope [56] using a 638-nm excitation laser. Fifty frames at an exposure time of 50 ms were recorded for at least nine field of views per sample.

### DNA-Points accumulation for imaging in nanoscale topography (PAINT)

For Aβ and AT8-positive tau super-resolution imaging, SiMPull coverslips were prepared as described above, but instead of the Alexa-Fluor-647-labelled imaging antibody, a DNA-labelled antibody was introduced [57, 58] (labelling protocol explained in ‘Reproducibility of findings in DS organoids’). After the washing steps, TetraSpeck microspheres (1:12,000 in TBS, 10 µL, Thermo Scientific; Cat. T7279) were introduced to each well for 10 min. The TetraSpeck solution was then removed, followed by a single wash with TBST, and a second PDMS gasket (Merck, GBL-103250-10EA) was stacked on the coverslip before introducing 4 µL of imaging strand (TGGTGGT- cy3B; atdbio) in TBS. Finally, the coverslip was sealed with another coverslip on top of the second PDMS gasket.

### Stochastic optical reconstruction microscopy (STORM)

For ASC-specks super-resolution imaging, SiMPull coverslips were prepared as described above. Three more PDMS gaskets were stacked together using nail polish and were afterwards filled with 15 µL STORM imaging solution. The stacked imaging chamber was sealed with a coverslip to avoid oxygen penetration. The coverslip was imaged using a 638-nm excitation laser for 4000 frames at an exposure time of 15 ms for three field of views per sample.

The imaging solution for STORM was prepared as reported [59]. Briefly, the following stock solutions were prepared: 0.1 M Tris supplemented with 20 mM NaCl, pH 8, filtered by 0.02 μm filter (VWR, cat. no. 516–1501), stored at 4 °C (2× imaging solution of STROM). 25% glucose, stored at 4 °C (2.5× imaging solution of STORM). 1 M cysteamine (Merck, cat. no. 30070) in 0.36 M HCl, stored at 4 °C for no more than 1 week (20× imaging solution of STORM). GOD buffer (24 mM PIPES, 4 mM MgCl_2_, 2 mM EGTA) at pH 6.8 and filtered by 0.02 μm filter, stored at 4 °C. 20 mg/mL glucose oxidase from Aspergillus niger (Merck, cat. no. G2133) in GOD buffer, centrifuge filtered with 0.22 μm filter (Merck, cat. no. UFC30GV0S), flash-frozen in liquid nitrogen, stored in −80 °C (40× imaging solution of STORM). 5 mg/mL catalase (Merck, cat. no. C40) in GOD buffer, centrifuge filtered with 0.22 μm filter (Merck, cat. no. UFC30GV0S), flash-frozen in liquid nitrogen, stored in −80 °C (125× imaging solution of STORM).

The final working imaging solution for STORM contains 0.5 mg/mL glucose oxidase, 40 μg/mL catalase, 50 mM cysteamine, and 10% glucose in 50 mM Tris supplemented with 10 mM NaCl at pH 8. This solution was prepared freshly and immediately before imaging.

### Antibody modifications

AL177 (for biotinylation) and 6E10 (for DNA conjugation) were functionalised using a SiteClick™ Antibody Azido Modification Kit (Invitrogen, Cat. No. S20026), according to the manufacturer’s instructions. Briefly, 200 μg of antibody was concentrated and buffer exchanged into the provided antibody preparation buffer. The antibody was then incubated overnight with β-galactosidase at 37 °C. On the next day, the reaction mixture was coupled to UDP-GalNAz using β-1,4-galactosyltransferase overnight at 30 °C. The mixture was then purified by Amicon spin filter (50 kDa MWCO, Merck, Cat. No. UFC505024). The concentration of the azido-modified antibody was determined by A280.

For biotinylation of AL177, DBCO-PEG4-biotin (Merck, Cat. No. 760749, Lot No. MKCN1219) was first dissolved in anhydrous DMSO at 10 mM. It was then selectively conjugated to the azido-modified antibodies. To the azido-modified antibody, 10 molar equivalents of DBCO-PEG4-biotin were introduced for SPAAC. After overnight incubation at 37 °C, excess DBCO-PEG4-biotin was removed by an ZebaTM Spin Desalting Column (40 kDa MWCO). The biotinylated antibody was then concentrated by an Amicon spin filter (50 kDa MWCO), and its concentration was determined by A280. The degree of labelling was determined using PierceTM Fluorescence Biotin Quantification Kit (Thermo Scoentific; Cat. 46610).

For DNA conjugation of 6E10, DBCO-modified oligonucleotides (10 molar equivalents; DBCO TEG-AAACCACCACCACCACCACCACCACCACCACCACCA; atdbio) were introduced to the azido-modified antibody, for copper-free strain-promoted click reaction (SPAAC) overnight at 37 °C. After that, the excess oligonucleotide was removed using an Amicon spin filter (100 kDa MWCO) and the concentration of antibody and degree of labelling were determined by A280 and A260/A280. The purity and degree of labelling were further confirmed using SDS-PAGE under reducing conditions.

### SiMoA

For the Aβ assay, SiMoA^®^ Homebrew carboxylated beads (Quanterix; Cat. No. 104006) were first functionalised with capture antibody 6E10, according to the manufacturer’s instructions. Briefly, the antibodies were first buffer-exchanged twice with the provided bead conjugation buffer, 25 mM MES pH 5.0, via Centrifugal Spin Filters (MWCO 50 kDa) as provided in the Homebrew 2.0 Development Kit (Quanterix, Cat. No. 101354). The concentration of the buffer-exchanged antibodies was monitored by A_280_. Meanwhile, 1 mL of the paramagnetic carboxylated beads (4.2 × 10^8^ beads) were washed three times with the provided bead wash buffer followed by three times with the bead conjugation buffer with the aid of the vortex and magnetic separator. EDC (Thermo Scientific, Cat. No. A35391) was dissolved in cold 25 mM MES pH 5.0 to give 10 mg/mL stock solution. After that, 9 µL of EDC solution was introduced to the washed beads and the reaction mixture was incubated on a mixer in a cold room for 30 min. The activated beads in the reaction mixture were washed once with cold 25 mM MES pH 5.0 with the aid of the vortex and magnetic separator. The buffer-changed antibodies (0.2 mg/mL, 300 µL) were then introduced to the activated beads. The reaction mixture was incubated on a mixer at 4 °C for 2 h. The conjugated beads were then washed twice with the provided bead wash buffer and blocked by the provided bead blocking buffer on a mixer at room temperature for 40 min. Finally, the blocked beads were washed once with the provided bead wash buffer and then once with the provided bead diluent. The beads were then dispersed in the bead diluent and stored at 4 °C until use. The coating efficiency of the beads was calculated by A_280_ of the supernatants before the 40-min blocking.

The beads coated with the 6E10 antibody were first washed three times with the provided bead diluent in the kit. Meanwhile, to each well on a conical bottom microplate (Quanterix), conditioned media samples were diluted with the provided sample diluent (5:95) to give 100 µL solution at the desired concentration. Then, 25 µL of the washed beads were introduced to each well to give the final-bead concentration as 2 × 10^7^ beads/mL. The samples and the beads were incubated on the plate shaker at 30 °C at 800 rpm for 30 min. The plate was then washed by the SiMoA washer with the provided buffers. Afterwards, the biotinylated detector antibodies 6E10, 11A50 (BioLegend; Cat. 805404), and 12F4 (BioLegend; Cat. 805504) were then diluted to 0.1 µg/mL with the provided Homebrew detector diluent and 100 µL of the diluted detectors were introduced to each well. The mixture was then incubated on the plate shaker at 30 °C with 800 rpm for 10 min. Similarly, the plate was then washed by the SiMoA washer. Meanwhile, the provided SBG concentrate was diluted to 50 pM with SBG diluent. To each well, 100 µL of the diluted SBG was introduced and the mixture was incubated on the plate shaker at 25 °C at 800 rpm for 10 min. Finally, the plate was washed by the SiMoA washer and loaded onto the SR-X™ Biomarker Detection System (Quanterix) together with the SiMoA disc, tips, and an equilibrated, well-shaken and opened RGP bottle.

The total tau assay (SiMoA tau advantage kit, Cat. No. 101552) was run following the assay instructions. In brief, conditioned media samples were diluted 1:200, incubated with the antibody-coated beads, and following the washing and detector antibody incubation steps loaded onto the SR-X™ Biomarker Detection System (Quanterix) together with the SiMoA disc, tips, and an equilibrated, well-shaken and opened RGP bottle.

### TXNIP ELISA

Thioredoxin-interacting protein (TXNIP) concentrations in the conditioned media samples were measured using colorimetric assays (antibodies.com; Cat. A76729) following the assay instructions. In brief, 50 μL of media sample from each condition was mixed with 50 μL of sample dilution buffer and loaded to a pre-coated 96-well plate in duplicates, alongside assay standards. Following a 90-min incubation at 37 °C, wells were washed twice and 100 µL of biotin-labelled antibody working solution was added to each well. Following a 60-min incubation at 37 °C, plate was washed three times and 100 µL of HRP-streptavidin conjugate working solution was added to each well. Following a 30-min incubation at 37 °C, the plate was washed five times and 90 µL TMB substrate solution was added to each well. Following a 15-min incubation at 37 °C, the reaction was stopped by adding 50 µL stop solution and A_450_ was measured.

### Data analyses

Media samples were encrypted with a unique identification code and during the experiments, the investigators were blinded to sample genotype. Diffraction limited imaging on SiMPull was performed as at least nine field of views (technical replicates), from which the data were averaged. Super-resolution imaging was performed as at least 3 field of views, from which the data were pooled. All ELISA and SiMoA assays were run in at least duplicates and the data were averaged. Diffraction-limited images were reconstructed and analysed using ComDet v.0.5.5 plugin for ImageJ and the super-resolution images were reconstructed, drift-corrected, and analysed using a software developed by our group [60]. G*Power software was used to estimate the difference needed between the DS or control organoid media samples to achieve enough power to reduce the Type 2 error rate below 0.05 with 3 independent experiments. The R Project Statistical Computing version 4.2.2 (2022-10-31)—“Innocent and Trusting” was used for all statistical analyses, the graphs (Mean ± SEM) were generated in GraphPad Prism 7.0a for Mac OS X, and the cartoon figures were created with BioRender.com. ANOVA’s with Type 2 sums of squares and Welch Two Sample *t* tests (2-tailed) were used to determine differences between the groups and corresponding 95% confidence intervals (CI) were reported. The datasets generated and analysed during this study are available from the corresponding author upon reasonable request.

## Results

### Study 1: Characterisation of soluble aggregates secreted by T21 (Down’s syndrome) and D21 (control) organoids

#### Schematic of approach

**Fig. 1:**
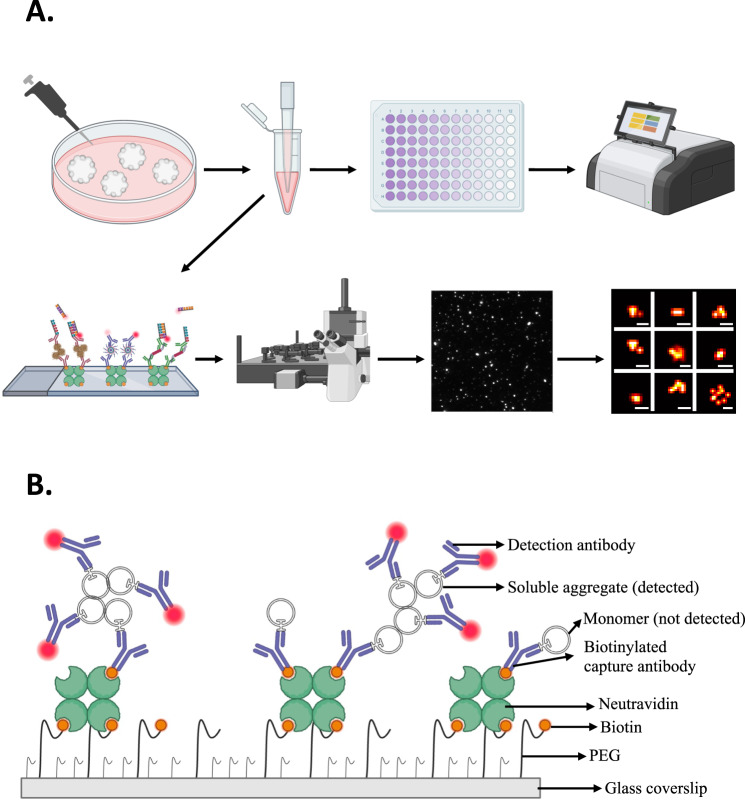
Schematic representation of the experimental design and single-molecule pulldown (SiMPull). **A** Conditioned media samples were collected from the organoids, aliquoted into LoBind Eppendorf tubes at ~20 µL samples, and stored at −80 °C until used. Glucose assay, SiMoA, and TXNIP ELISA were performed as plate-based assays. For diffraction-limited and super-resolution imaging, SiMPull, TIRF microscopy, DNA-PAINT, and STORM methodology were used. **B** Glass coverslips were pacified with PEG’s and incubated with biotinylated capture antibody. Samples were incubated overnight and imaged using dye or DNA-labelled imaging antibodies. Using the same antibody pair to capture and image ensured the detection of dimers and larger species (no monomers) as the single epitope on a monomer was saturated by the capture antibody.

The method is based on collecting and freezing the conditioned media from wells containing a pool of 12–16 isogenic organoids (Fig. 1A). These aliquots are stored and then defrosted for analysis using a modified SiMPull method [61]. Antibodies are attached to a PEG surface using neutravidin-biotin for selective capture of the aggregate of interest. These aggregates are then imaged using a fluorophore-labelled antibody. Since every monomer has a single epitope for a given monoclonal antibody, using the same antibody to capture (biotinylated) and detect (dye conjugated) ensures the detection of aggregates (dimers and larger species) and no monomers (Fig. 1B, Supplementary Fig. 3A, B), with low levels of unspecific binding and noise from unconditioned media (Supplemental Fig. 3C–F). We used monoclonal antibodies 6E10 for detection of Aβ aggregates and AT8 for detection of phosphorylated tau (pTau) aggregates. We first used diffraction-limited imaging to detect the number of aggregates on the surface as a measure of the relative concentration of aggregates in the media and then used super-resolution imaging to determine the size, area, and eccentricity of the aggregates with a resolution limit of 20 nm [56]. We also detected ASC-specks in the media, using a pair of antibodies to measure the extent of inflammasome activation. Quantifying these ASC-specks have been shown to be a reliable marker of inflammasome activation [62] (Lobanova et al., in preparation). TXNIP release was also quantified as a measure of oxidative stress, using an ELISA assay.

#### Utilisation of glucose consumption in culture media to normalise for the amount of living cells in organoid cultures

**Fig. 2:**
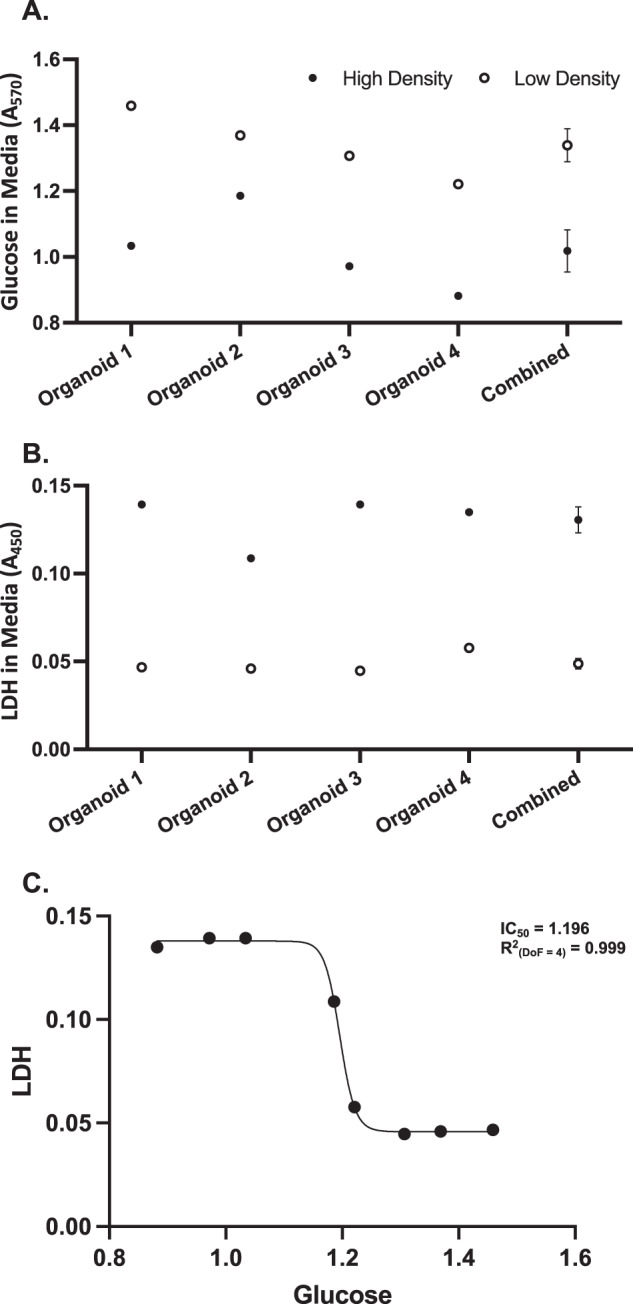
Validation of the glucose consumption assay as a nornalisation tool. High-density samples contained 8–11 organoids, meanwhile the low-density samples contained two organoids. Conditioned media samples were collected at 100 days in vitro. **A** Amount of glucose left in the conditioned media samples after 4-days in vitro. All samples were tested in two technical replicates and the means were plotted. **B** Amount of lactate dehydrogenase (LDH) accumulated in the conditioned media samples after 4-days in-vitro. All samples were tested in two technical replicates and the means were plotted. **C** LDH levels were plotted against glucose levels and a sigmoid curve was fitted. The IC_50_ value of the curve was 1.196 with a logIC_50_ of 0.078, and hillslope of −92.020. The *R*^2^ value of the curve was 0.999.

One major problem of longitudinal studies with conditioned media samples is the positive correlation between the number of living cells in the sample and the amount of the target of interest released to the media. Thus, it is critical to normalise the media samples with the number of living cells at all time points. While the experiment is designed to start with an identical number of organoids per well for all compared genotypes/conditions, (all starting from the same human iPSC cell number), organoids are known to vary in size, fuse during culture, and/or grow at variable rates. Counting the resulting numbers of cells in organoids would require dissociation, making it a non-suitable method for longitudinal studies. We have here developed a novel assay, based on determining the amount of glucose (the primary source of metabolic energy) left in the media, as a function of consumption by viable cells. We validated this method by measuring the glucose and LDH levels in the condition media of four different organoid samples, each collected from organoids with high (8–11 organoids per well) or low cell density (two organoids per well). While the amount glucose left in the media was significantly lower in the samples with high cell density (*t*_5.68_ = 3.94, *p* = 0.008, *d* = 3.31, Fig. 2A), the amount of LDH in the media was significantly higher for these samples (*t*_3.97_ = 10.28, *p* < 0.001, *d* = 10.32, Fig. 2B). Moreover, there was a significant negative correlation between the LDH and glucose values (*r*_6.00_ = −0.918, *p* = 0.001, Fig. 2C), collectively suggesting that organoid samples with higher cell density are consuming more glucose from the media, making the amount of leftover glucose a valid measure to predict and normalise for the number of cells. Thus, this method was used to normalise the results for aggregate quantification presented below.

#### Chromosome 21 trisomy increases the concentration of soluble Aβ aggregates released by the organoids

**Fig. 3:**
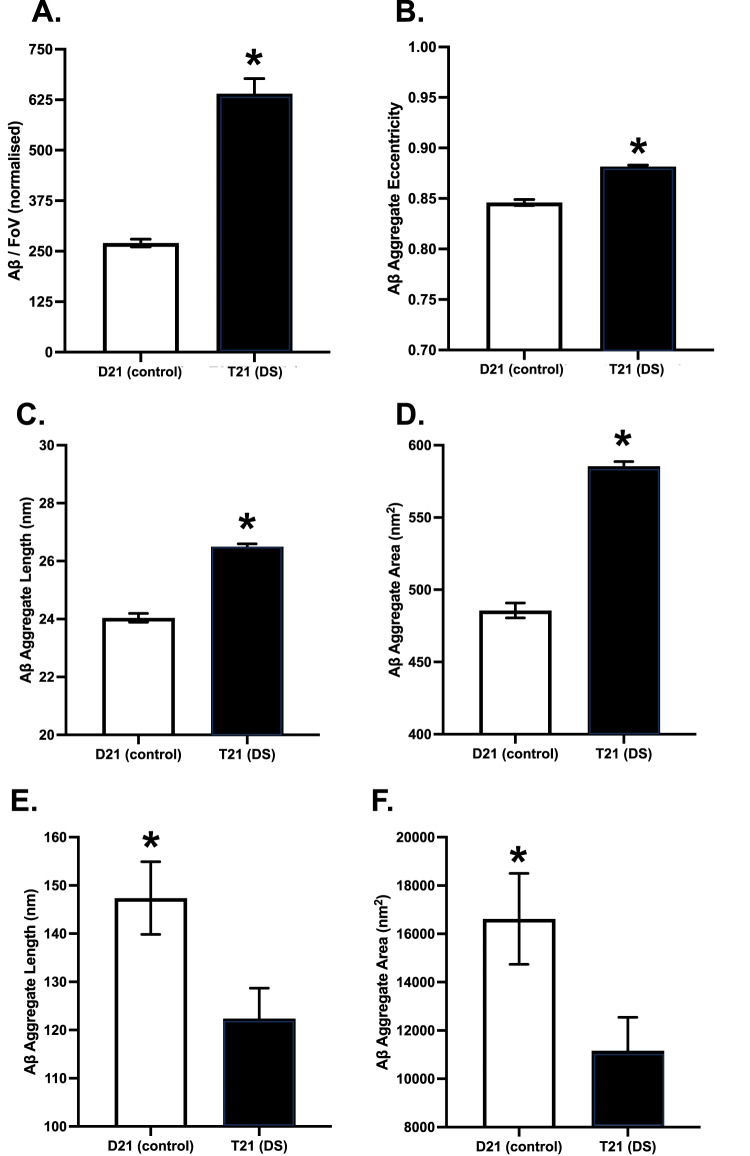
Characterising beta-amyloid (Aβ) aggregates in the conditioned media samples using diffraction-limited and super-resolution (DNA-PAINT) microscopy with 6E10 antibody. **A** Mean (±SEM) number of Aβ aggregates in each field of view, averaged over three fields of views in two technical replicates, normalised by glucose consumption. **B** Mean (±SEM) Aβ aggregate eccentricity, in which 0 indicates perfectly circular and 1 indicates perfectly flat aggregates. **C** Mean (±SEM) Aβ aggregate predicted length and **D** area for aggregates below 4000 nm^2^. **E** Mean (±SEM) Aβ aggregate predicted length and **F** area for aggregates above 4000 nm^2^.

We used SiMPull with TIRF microscopy to quantify the amount of Aβ aggregates in the T21 and D21 organoids between 84 and 150 DIV. Since there were no significant time-point effects within the T21 and D21 organoids, data were pooled and further analysed for genotype effects. Although both the T21 and D21 organoids produced soluble Aβ aggregates, the T21 organoids released significantly more Aβ aggregates to the media than the isogenic D21 control organoids, by a factor of 2.5-fold (*t*_80.39_ = 9.65, *p* < 0.001, CI_95_ = 293.59, 446.19; Fig. 3A).

#### Chromosome 21 trisomy increases the monomeric Aβ concentration released by the organoids

Following the quantification of soluble Aβ aggregates using SiMPull, we further validated these results with a super-sensitive single-molecule array (SiMoA) assay [63] using the same (6E10) antibody to capture and detect the aggregates. Then, we combined the 6E10 capture antibody with the Aβ_40_ specific 11A50 and Aβ_42_ specific 12F4, to measure the concentration of these specific Aβ species. Since different antibodies were used to capture and detect in these later experiments, the total monomer concentration was also detected, showing the total Aβ production in the organoids.

**Fig. 4:**
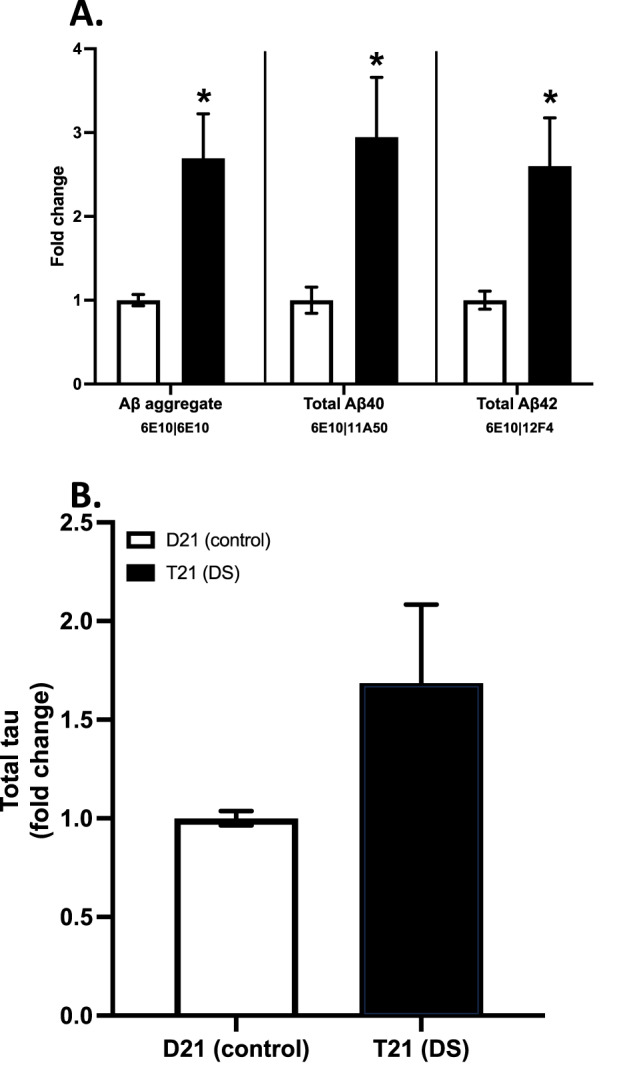
Quantification of Aβ and tau in conditioned media samples using SiMoA, normalised to glucose consumption values. Results presented as fold-change in comparison to D21 (control) organoids. **A** Fold-change total Aβ aggregates quantified using 6E10 capture and detection antibodies, as well as total Aβ40 (monomeric and aggregated) and total Aβ42 (monomeric and aggregated) quantified using 11A50 and 12F4 detection antibodies, respectively. **B** Fold-change total tau (monomeric and aggregated) quantified using SiMoA total tau assay kit.

Agreeing with the SiMPull results, T21 organoid media samples contained ~2.7-fold higher amounts of Aβ than the D21 organoid media (*t*_7.22_ = 3.17, *p* = 0.015, CI_95_ = 0.58, 3.91; Fig. 4A). Moreover, the T21 organoids also released ~3-fold more total (monomeric and aggregated) Aβ_40_ (*t*_7.66_ = 2.68, *p* = 0.029, CI_95_ = 0.82, 11.60; Fig. 4A) as well as ~2.6-fold more Aβ_42_ (*t*_7.48_ = 2.74, *p* = 0.027, CI_95_ = 1.95, 24.66; Fig. 4A) to the media than the D21 organoids. Meanwhile, the ratio of Aβ_42_ to Aβ_42_ + _40_, did not differ between the organoids (*t*_12.49_ = 1.32, *p* = 0.210).

#### Super-resolution imaging of soluble Aβ aggregates shows detectable differences in aggregate size and eccentricity

**Fig. 5:**
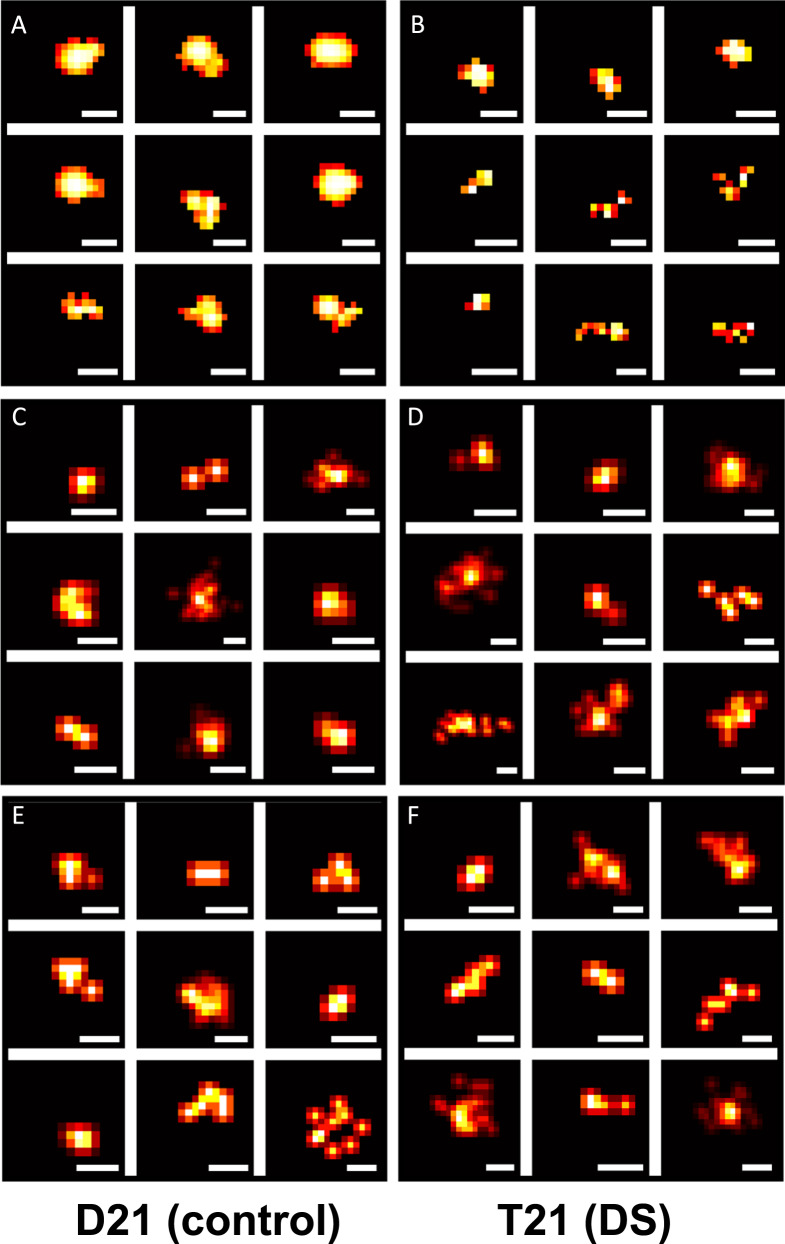
Representitive super-resolved images of Aβ, pTau, and ASC-speck detected in cerebral organoid conditioned media. Representative images of **A**, **B**. Aβ, **C**, **D**. AT8-positive tau, and **E**, **F**. ASC-speck aggregates, from DNA-PAINT and STORM microscopy, reconstructed by ACT software. **A**, **C**, **E** are D21 (control), **B**, **D**, **F** are T21 (DS) and the scale bar is 50 nm.

After quantifying soluble Aβ aggregates in the media samples, we characterised their morphology in terms of length, area, and eccentricity, using DNA-Points Accumulation for Imaging in Nanoscale Topography (PAINT; see Fig. 5A, B for representative images). We observed a range of aggregates from 20 nm in length to over 100 nm, but the majority of the aggregates formed were small with an average length of 27 nm, which did not differ between the T21 and D21 organoids (*t*_14261_ = 1.12, *p* = 0.263, CI_95_ = −0.99, 0.27). Meanwhile, the aggregates released by the D21 organoids had an overall larger mean area (*t*_12162_ = 3.64, *p* < 0.001, CI_95_ = 88.59, 295.41). However, the cumulative length and area distribution plots indicated that the majority of the aggregates released by the T21 organoids are larger than the ones released by the control organoids, but the very few, extremely large aggregates released by the control organoids skew the results. In order to investigate this, we separated the results by area as the aggregates with an area less or more than 4000 nm^2^. By this size grouping, 98.5% of all aggregates (98.8 for T21 and 97.4 for control) were in the group smaller than 4000 nm^2^. Within this group, the average length (24.04 ± 0.15 nm for D21, 26.50 ± 0.10 nm for T21, *t*_19282_ = 13.54, *p* < 0.001, CI_95_ = 2.10, 2.81; Fig. 3C; Supplementary Fig. 4A) and the area (485.71 ± 5.15 nm^2^ for D21, 585.52 ± 3.08 nm^2^ for T21, *t*_18495_ = 16.64, *p* < 0.001, CI_95_ = 88.05, 111.56; Fig. 3D) of the Aβ aggregates released by the T21 organoids were larger than the aggregates released by the control organoids. Indicated by higher eccentricity values (0.85 ± 0.003 for D21, 0.88 ± 0.001 for T21, *t*_15242_ = 10.80, *p* < 0.001, CI_95_ = 0.03, 0.04), the aggregates released by the T21 organoids were also more fibrillar (less circular; Fig. 3B; Supplementary Fig. 4B). On the other hand, for the group of aggregates larger than 4000 nm^2^, constituting 1.5% of the aggregates, the average length (147.38 ± 7.53 nm for D21, 122.38 ± 6.30 nm for T21, *t*_534.5_ = 2.55, *p* = 0.011, CI_95_ = 5.71, 44.29; Fig. 3E; Supplementary Fig. 4C) and the area (16,620.40 ± 1880.30 nm^2^ for D21, 11167.87 ± 1376.16 nm^2^ for T21, *t*_490.9_ = 2.34, *p* = 0.020, CI_95_ = 874.3435, 10030.7289; Fig. 3F; Supplemental Fig. 4D) of the Aβ aggregates released by the T21 organoids were smaller than the aggregates released by the control organoids. Eccentricity did not differ between the samples in this group (0.84 ± 0.008 for D21, 0.85 ± 0.007 for T21, *t*_542.0_ = 0.63, *p* = 0.532, CI_95_ = −0.03, 0.02).

Overall, these experiments show that in the T21 organoids compared to D21, a stable higher production of Aβ correlates with a higher number of soluble aggregates, that are in most cases larger and more fibrillar. However, some extremely large aggregates are released from the control organoids.

#### Chromosome 21 triplication does not increase total tau levels but increases phosphorylated tau aggregate release without altering their morphology

Similar to Aβ, total (monomeric and aggregate) tau levels were measured in the conditioned media samples using SiMoA. According to this assay, total tau in the media did not differ between T21 and D21 organoids (*t*_4.07_ = 1.71, *p* = 0.162, CI_95_ = −85.88, 20.22; Fig. 4B).

**Fig. 6:**
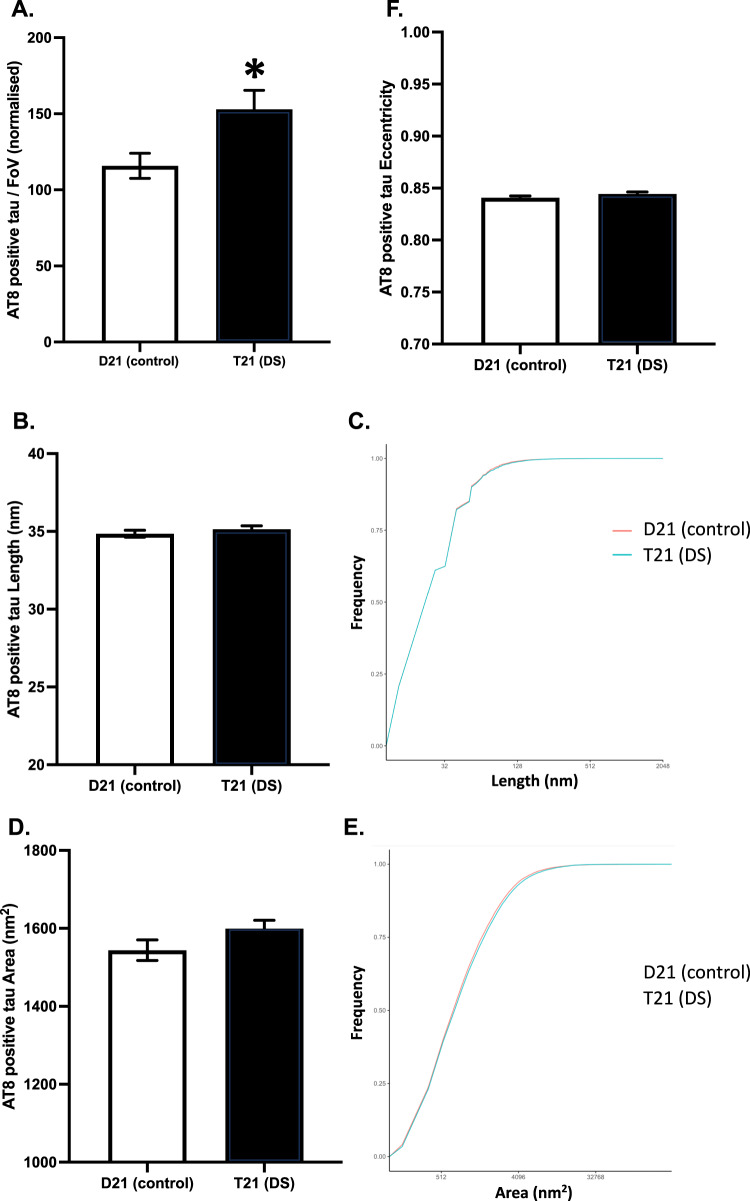
Characterising AT8-positive tau aggregates in the conditioned media samples using diffraction-limited and super-resolution (DNA-PAINT) microscopy with AT8 antibody. **A** Mean (±SEM) number of tau aggregates in each field of view, averaged over three fields of views in two technical replicates, normalised by glucose consumption. **B** Mean (±SEM) tau aggregate predicted length and **C** the cumulative predicted length distribution. **D** Mean (±SEM) Aβ aggregate area, and **E** The cumulative area distribution. **F** Mean (±SEM) tau aggregate eccentricity, in which 0 indicates perfectly circular and 1 indicates perfectly flat aggregates.

On the other hand, SiMPull demonstrated that T21 organoids released 1.3-fold more AT8-positive tau aggregates to the media than the D21 organoids (*t*_123.43_ = 2.49, *p* = 0.014, CI_95_ = 7.66, 66.65; Fig. 6A). Meanwhile, the average length (*t*_34423_ = 0.92, *p* = 0.358, CI_95_ = −0.90, 0.32; Fig. 6B, C), area (*t*_33599_ = 1.61, *p* = 0.108, CI_95_ = −122.00, 12.00; Fig. 6D, E), or eccentricity (*t*_34375_ = 1.46, *p* = 0.144, CI_95_ = −0.01, 0.01; Fig. 6F) of these AT8-positive tau aggregates did not differ between the genotypes (see Fig. 5C, D for representative images).

Overall, these data show that while chr21 trisomy does not increase tau expression, it increases the release of soluble aggregates of phosphorylated tau by a factor of ~1.3-fold consistent with the T21 genetic condition. Importantly, both models secreted aggregates of AT8 positive tau, which were mainly small, on average around 25–27 nm in length, but there were some aggregates larger than 100 nm.

#### Chromosome 21 trisomy increases the number of ASC-specks released by the organoids without altering speck morphology and increases TXNIP concentration

An essential component of the amyloid-cascade hypothesis is the promotion of tau pathology, which was reproducible by our organoid system, on the level of pathophysiologogy. Several possible links between Aβ aggregation and tau hyperphosphorylation have been suggested, among them the inflammasome activation and increased oxidative stress. We measured ASC-specks (see Fig. 5E, F for representative images) and TXNIP in the conditioned media samples as markers of these pathological mechanisms.

**Fig. 7:**
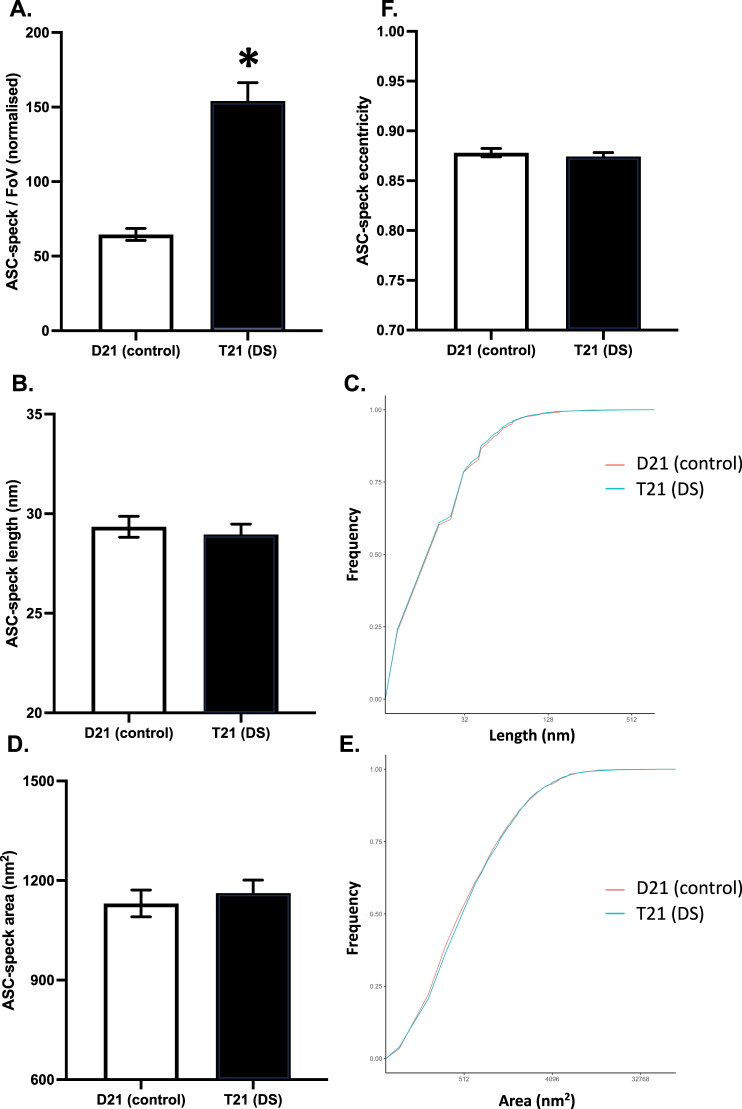
Characterising ASC-specks in the conditioned media samples using diffraction-limited and super-resolution (STORM) microscopy with AL177. **A**. Mean (±SEM) number of ASC-specks in each field of view, averaged over three fields of views in two technical replicates, normalised by glucose consumption. **B** Mean (±SEM) ASC-speck predicted length and **C** The cumulative predicted length distribution. **D**. Mean (±SEM) ASC-speck area, and **E** The cumulative area distribution. **F** Mean (±SEM) Aβ aggregate eccentricity, in which 0 indicates perfectly circular and 1 indicates perfectly flat aggregates.

**Fig. 8:**
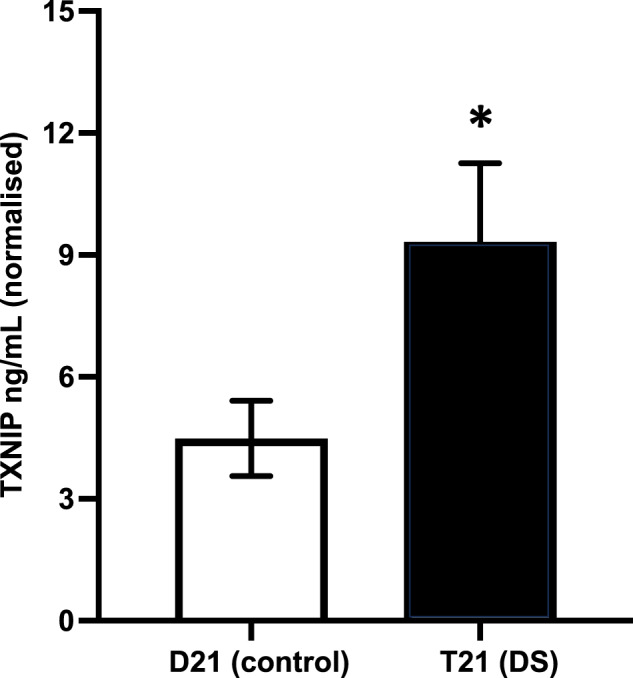
Trisomy 21 increases the production of TXNIP in cerebral organoid conditioned media. Mean (+SEM) levels of TXNIP (ng/mL) measured using ELISA in the conditioned media samples, normalised by the glucose consumption assay.

While the T21 organoids released significantly more ASC-specks to the media than the D21 organoids (*t*_173.58_ = 6.93, *p* < 0.001, CI_95_ = 63.99, 114.98; Fig. 7A), the length (*t*_5820.1_ = 0.52, *p* = 0.604, CI_95_ = −1.06, 1.83; Fig. 7B, C), area (*t*_5809.5_ = 0.56, *p* = 0.577, CI_95_ = −141.45, 78.72; Fig. 7D, E), or eccentricity (*t*_5728.4_ = 0.66, *p* = 0.507, CI_95_ = -0.01, 0.02; Fig. 7F) of these specks did not differ. Moreover, T21 organoids released more TXNIP to the media than the D21 organoids (*t*_9.99_ = 2.26, *p* = 0.048, CI_95_ = 0.06, 9.62; Fig. 8).

Together, these results show that chr21 triplication causes significantly elevated inflammasome formation and elevated oxidative stress levels. The potential link between these markers and pathological tau aggregation could, therefore, be studied in a human cerebral organoid system.

### Study 2: Testing the reproducibility of the model by replicating the key findings in two additional repeat experiments

#### Increased concentration of soluble Aβ aggregates is conserved in additional clones and experiments

**Fig. 9:**
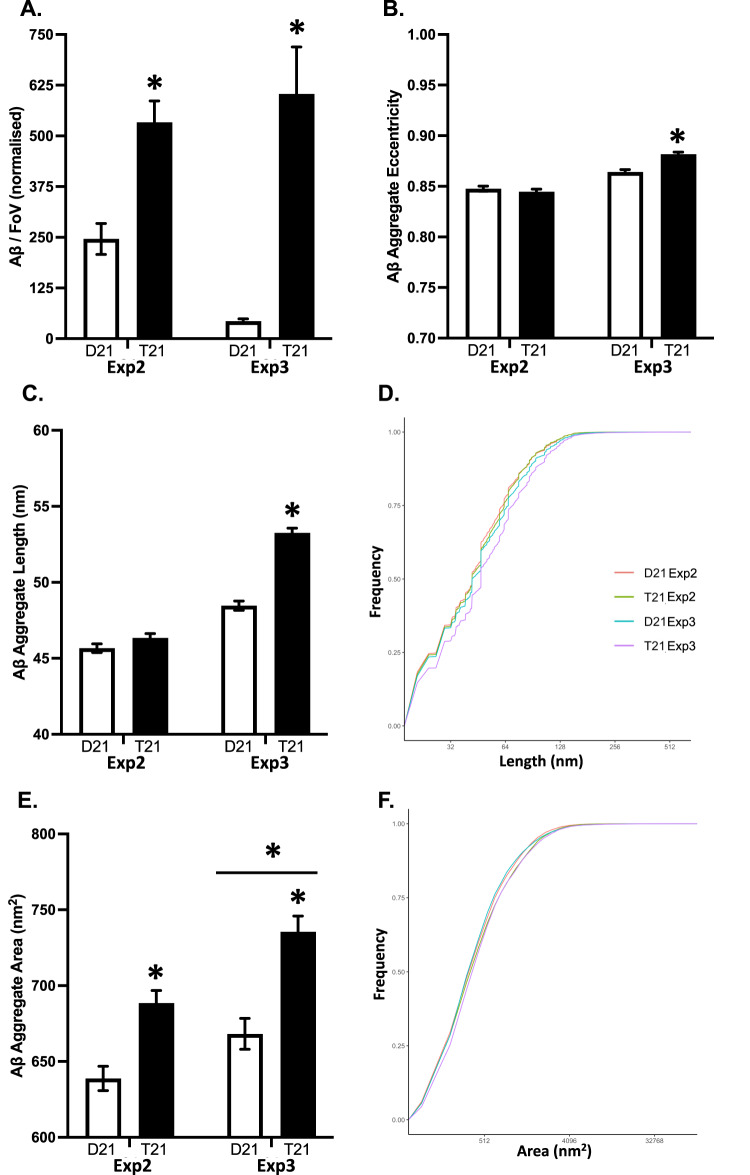
Characterising beta-amyloid (Aβ) aggregates in the conditioned media samples from the additional experiments (Exp2 and Exp3) using diffraction-limited and super-resolution (DNA-PAINT) microscopy with 6E10 antibody. **A** Mean (±SEM) number of Aβ aggregates in each field of view, averaged over three fields of views in two technical replicates, normalised by glucose consumption. **B** Mean (±SEM) Aβ aggregate eccentricity, in which 0 indicates perfectly circular and 1 indicates perfectly flat aggregates. **C** Mean (±SEM) Aβ aggregate predicted length and **D** the cumulative predicted length distribution. **E** Mean (±SEM) Aβ aggregate area, and **F** the cumulative area distribution.

We performed two additional experiments (henceforth referred as Exp2 and Exp3) each started from undifferentiated iPSCs, prior to differentiation to organoids. These were performed by different experimenters, in different laboratories, at different times, and using additional, different iPSC clones (independent reprogramming events) from the same mosaic DS individual. Agreeing with the first study, the T21 organoids from both additional experiments produced more soluble Aβ aggregates than the D21 organoids (AIC = 3087.3, *F* = 36.69, *p* < 0.001). While there was no significant difference between the experiments (AIC = 3053.5, *F* = 0.76, *p* = 0.385), the genotype (T21 vs D21) difference was more pronounced in Exp3 (2.2 fold vs 13.9 fold; Fig. 9A).

#### Soluble Aβ aggregates released by the different clones, in separate experiments share similar morphological characteristics

Then, we performed DNA-PAINT imaging on the soluble Aβ aggregates, to determine their length, area, and shape. Overall, the aggregates were larger in the T21 organoids in both repeat clones (AIC = 327282.0, F = 91.35, *p* < 0.001), agreeing with the initial study—once the few extremenly large aggregtes in the D21 samples were removed. However, the genotype difference only reached significance for the third experiment (Exp2: CI_95_ = −1.47, 0.12; Exp3 CI_95_ = 3.93, 5.65; Fig. 9C, D). For the average aggregate area, there were main effects of genotype (AIC = 651394.0, F = 38.55, *p* < 0.001) and experiment (AIC = 651372.0, F = 16.11, *p* < 0.001). While the area of the Aβ aggregates released by the T21 organoids were larger than the aggregates released by the D21, overall, the aggregates released in Exp3 were larger for both genotypes (Fig. 9E, F). For aggregate eccentricity, there was a genotype difference in Exp3, agreeing with the initial study as the T21 aggregates were more fibrillar than the D21 aggregates (CI_95_ = 0.01, 0.02), but not in Exp2 (CI_95_ = −0.01, 0.01), resulting in a genotype by experiment interaction (AIC = 127101.0, F = 18.59, *p* < 0.001; Fig. 9B).

#### Increased phosphorylated tau aggregates were reproduced in two of the three experiments

**Fig. 10:**
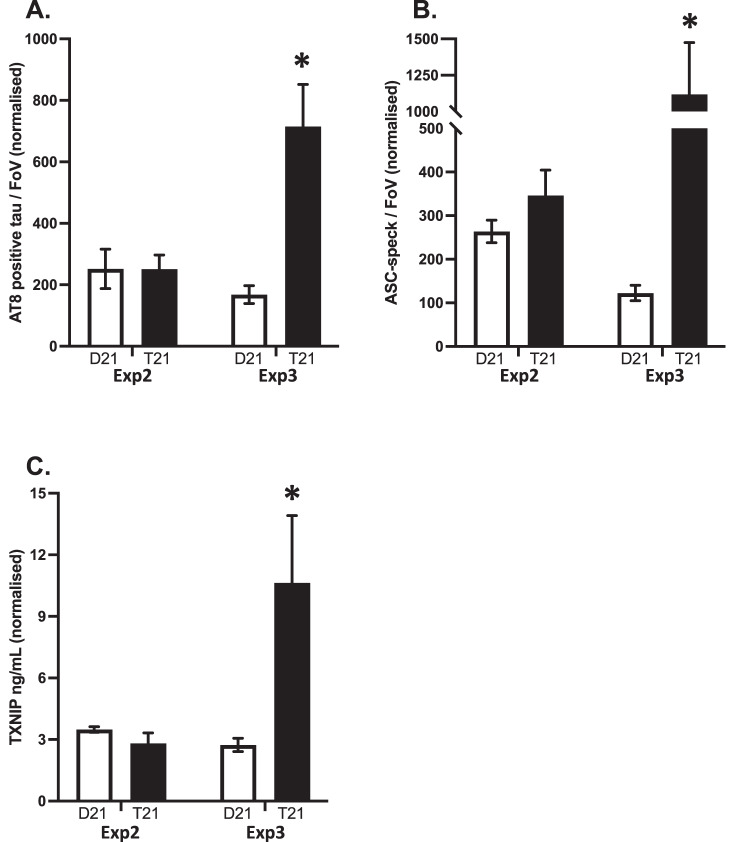
Reproducibility of soluble aggregate quantitation in cerebral organoid conditioned media in independent experiments starting from clonally different undifferentiated iPSC. Quantifying (Mean ± SEM) **A** AT8-positive tau aggregates, **B** ASC-specks, and **C** TXNIP in media samples from the additional experiments 2 and 3, normalised to glucose consumption values.

After soluble Aβ aggregate pathology, we looked at AT8-positive tau aggregate levels in the additional clones. There was a genotype by experiment interaction (AIC = 2315.0, F = 8.9416, *p* = 0.003), as the increased AT8-positive tau levels observed in the initial study were only reproduced in Exp3 (Exp2: CI_95_ = −156.49, 158.01; Exp3: CI_95_ = 269.81, 824.27; Fig. 10A).

#### Increased inflammation and oxidative stress segregate with organoids that also produce soluble AT8-positive tau aggregates

While ASC levels were increased in the T21 organoids in both experiments (AIC = 2633.8, *F* = 8.31, *p* = 0.004), the difference was significant only for Exp3 (Exp2: CI_95_ = −209.63, 44.50; Exp3: CI_95_ = 285.92, 1704.60; Fig. 10B). Similarly, while TXNIP levels were higher in Exp3 (CI_95_ = 5.51, 152.38), they did not differ in Exp2 (CI_95_ = −6.12, 19.48; Fig. 10C).

## Discussion

AD is the most common cause of dementia and there is a need for validated and reliable models to understand the pathogenesis and test novel therapeutics. While various mouse models exist, producing human Aβ and tau, growing evidence suggests the involvement of numerous other proteins in the pathogenesis of AD [64], emphasising the importance of human-derived models. Cerebral organoids generated from individuals with DS could potentially be used as models of AD to investigate the amyloid cascade hypothesis, given the relationship between chr21 trisomy and AD. AD-like histopathology has been observed in organoids from multiple individuals with DS, and reproduced in several independent studies [43, 65, 66], and the increased secretion of Aβ peptides by T21 organoids was robustly reproducible in independent clones of multiple isogenic iPSC models [43, 66, 67]. However, the ability to generate reproducible quantitative data on more subtle phenotypes from organoid experiments remains a challenge in the field, due to high variability between experiments [67]. Here, using single-molecule detection techniques for soluble aggregates (Fig. 1, Supplemental Fig. 3), we first characterised the organoid conditioned media of single T21 and D21 iPSC clones from an isogenic DS model [52] (see also Supplemental Fig. 1) which did not show Aβ plaque and tau tangle histopathologies [43] (see also Supplemental Fig. 2). Then, we tested the reproducibility of the findings in two separate experiments (each starting de-novo from undifferentiated iPSCs). In both of these experiments the disomic and the trisomic line were different clones compared to the first experiment. Additionally, these two repeat experiment shared the same disomic clone, but used different trisomic clones. With the current sample size, at least 20 aggregates per field of view between the ASC-specks secreted by the T21 and D21 organoids was necessary to detect a significant difference, with an alpha value set to 0.05. For TXNIP the difference needed to be at least 2 ng/mL.

### Glucose consumption as a normalisation parameter

Regardless of sample type and analysis methodology, normalising is an essential step in studies aiming to quantify targets of interest in biological samples. When working with post-mortem tissue or cell lysate samples, this is done to control for the efficiency of homogenisation and protein collection, often using methods such as nanodrop or bicinchoninic acid (BCA) assays, meanwhile in studies quantifying gene expression, such as qPCR, the activity levels of the cells are controlled by normalising to house-keeping genes. For studies using conditioned media samples such as the current one, normalising to the number of living cells is necessary, as the target of interest in the media may change based on the number of cells releasing it, confounding the amount of release from individual cells. While the number of living cells can be determined by counting them, this requires dissociation and therefore destruction of the organoids, which is not possible in longitudinal studies. Since cell death is cumulative, methods such as LDH assay are not reliable over multiple time points. In this study, we developed and validated a novel glucose consumption assay (see Fig. 2), which to the best of our knowledge, is the first reliable method of normalising conditioned media samples collected longitudinally from cerebral organoids. Neurons, unlike most other cell types, can exclusively utilise glucose as the source of energy [68, 69] making the glucose consumption assay a highly appropriate measurement for the cerebral organoids, as they contain >90% neurons, with a very small percentage of astrocytes [43], and a nearly total lack of other brain cell types.

### Both control and T21 organoids produce soluble aggregates of Aβ

Using the glucose consumption assay to normalise the aggregate counts in conditioned media samples, we first compared the amount of soluble Aβ aggregates released from T21 and isogenic control D21 organoids. While the T21 organoids released significantly higher amounts of soluble Aβ aggregates than the control organoids throughout the experiment, the rate of release was stable over time. Moreover, we were able to detect soluble Aβ aggregates in the T21 media at the earliest time point (DIV 84), while the Aβ plaques in this line could not be detected within the timespan of 150 DIV [43]. Meanwhile, organoids generated from other, genotypically different DS individuals, showed flagrant amyloid plaques at DIV 100 [43]. This demonstrates the proof of principle of our ability to detect the formation and accumulation of soluble Aβ aggregates, that precede insoluble plaque formation. These results agree with findings in individuals with DS, as they show higher Aβ loads starting during foetal brain development and continuing throughout the lifetime, during which plaque pathology follows oligomer accumulation [70–73], thus our results of soluble aggregate detection preceding plaques indicate high external validity.

Remarkably, albeit less than the T21 organoids, the isogenic control organoids with two copies of chr21 also produced and released soluble Aβ aggregates (Figs. 3, 5, 9), which contradicts the hypotheses of Aβ aggregate production only being seen in pathological conditions such as AD and DS, and supports the idea of these conditions being diseases of shifted balance between Aβ production and clearance, leading to the formation and accumulation of more toxic species [74, 75]. Indeed, our earlier studies showed a significant size and shape difference between Aβ aggregates from AD patients and controls [50]. To investigate if the Aβ aggregates released from T21 organoids differ from the control organoids in terms of their morphology, we super-resolved the aggregates beyond the diffraction-limit of light, using DNA-PAINT. While the length, area, and eccentricity of the aggregates released by either the T21 or D21 organoids did not change over the time course of the experiment, the Aβ aggregates released by the T21 organoids were different from the ones released by the control organoids. While the average length was the same, the aggregates released by the T21 organoids had a different size distribution compared to the aggregates released by the D21 organoids. In particular, the T21 organoids had a higher proportion of longer (30–100 nm; Fig. 3C) and larger (300-2000 nm^2^; Fig. 3F) aggregates. However the extremely small proportion of very large aggregates (>4000 nm^2^) were predominantly released by the D21 organoids. This suggests that the T21 neurons are exposed to a much higher concentration of presumably toxic soluble aggregates, long before the formation of microscopically visible plaque-like structures, due to the increased rate of Aβ production and presumably because the longer, more fibrillar aggregates are less effectively cleared and accumulate into plaques. On the other hand, the D21 organoids, while producing fewer soluble aggregates, seem also able to rapidly sequester those into fewer, much larger super-aggregates (>4000 nm^2^), which in turn may reduce the concentration and time of exposure of neurons to their toxicity [51], and possibly provide insight for the presence of Aβ plaques in cognitively intact individuals.

### Soluble pTau aggregates

Hyperphosphorylation of tau protein, which causes its detachment from the microtubules and form intra-neuronal aggregates is a common pathology seen in AD. However, the relationship between Aβ aggregation and tau hyperphosphorylation is not fully understood [23, 76]. The existence of AT8-positive, hyperphosphorylated tau aggregates in DS brains and biofluids [77, 78] is considerable support for the amyloid-cascade hypothesis, given the role of APP over-production and Aβ accumulation in the pathogenesis of the disease. In this study, we showed an increase in AT8-positive soluble tau aggregates in the T21 organoid media samples, even though the release dynamics were stable over the time-course of the experiment and the morphology of the aggregates did not differ between T21 and control organoids. We have previously shown that organoids produced from this T21 line did not show a significant histological presence of pathologically conformed tau inside neurons by DIV 100 [43]. The detection of a higher amount of soluble tau aggregates in T21 organoid-conditioned media, is a proof of principle that overproduction of phosphorylated tau aggregates precedes the detection of large intra-neuronal tangle-like pathologically conformed tau formations.

Remarkably, the total amount of tau did not differ between the T21 and D21 organoids, showing that the pathological tau aggregation promoted by Aβ accumulation is downstream of tau expression. It has been suggested that immune activity and inflammasome formation can be a link between Aβ accumulation and tau pathology [79] via the kynurenine pathway, as NLRP3 activation can increase the expression of indoleamine 2, 3-dioxygenase (IDO) [80] which in turn increases quinolinic acid, promoting tau phosphorylation in neurons [81, 82]. Another possible mechanism is increased oxidative stress [83, 84]. Aβ_42_ can directly bind to the promoter region of the thioredoxin-interacting protein (*TXNIP*) gene and increase its expression in the cerebrum [85] which then increases reactive oxygen species (ROS) levels by inhibiting thioredoxin (TRX) [86] and increase tau phosphorylation via the ROS activated MAPK pathway [87]. To see if markers representing either of these mechanisms showed any correlation with increased AT8-positive soluble tau aggregate levels in the T21 organoids we measured inflammasome ASC-specks and TXNIP in the organoid conditioned media samples.

### ASC-specks

Along with increased Aβ accumulation, another shared pathology between DS and AD is the dysregulation of the immune system and inflammasome formation [88–91]. As we have shown previously, larger Aβ aggregates may be more inflammatory, leading to inflammasome formation, while smaller aggregates lack this type of toxicity [50, 51]. Inflammasomes are protein complexes made up of a sensor, an adaptor, and the inflammatory protease caspase-1 [92]. Studies have demonstrated that soluble Aβ can increase the expression of NLRP3, through the assembly of a toll-like receptor (TLR) 4 and 6 heterodimers [93, 94]. More recently, it has also been shown that oligomeric and fibrillar Aβ directly interacts with cytosolic NLRP3 to promote ASC binding and inflammasome formation [95, 96]. Although we detected ASC-specks in both T21 and D21 conditioned media samples, the levels were significantly higher in the T21 samples (Figs. 7, 10). This increased inflammasome formation is in line with the findings of De et al. [51] and shows an additional new in-vitro phenotype in cerebral organoids, closely mimicking that of human brains with AD.

### TXNIP

Oxidative stress and damage is another common feature of AD and DS [97–99]. Mitochondrial dysfunction leads to higher ROS formation, which in turn can bind to proteins and DNA, causing damage. TRX decreases ROS by binding to the oxidated species and reducing them, however TXNIP promotes ROS by binding to and inhibiting TRX [86]. We found elevated levels of TXNIP in the media of T21 organoids (Figs. 8, 10), along with increased inflammation, mitochondrial dysfuntion [52] and elevated oxidative stress caused by chr21 triplication and Aβ aggregation. Additionally, Aβ was shown to increase the levels of TXNIP in neuronal cells [100], and TXNIP-associated NLRP3 inflammasome was found activated in the brain of AD patients [101]. Therefore, the finding of elevated levels of TXNIP in media from our organoids, adds additional evidence that this organoid model recapitulates several authentic elements of the AD pathogenic process relevant to events in human brain.

### Reproducibility of findings in DS organoids

One of the main proposed limitations for the use of organoid models is the high variability between individual organoids, clones and experiments [45, 102]. Slight differences between the protocols used to develop the organoids or stochastic events such as variable growth speed, rupture and fusion of individual organoids during culturing can lead to changes in their phenotype [103]. In order to minimise the variation between individual organoids, our experimental design always used a pool of 12-16 organoids grown together in the same dish as a unit (Supplemental Fig. 1). Additionally, we invented and validated a method to normalise any measurements from a pool of live organoids to the total quantity of live neurons in such a pool, using the glucose consumption measurement (Fig. 1). Using this set up, here we were able to fully reproduce all significant differences found in the first part of the study (increased soluble Aβ aggregates with differences in structure, AT8-positive tau pathology, elevated ASC-specks and TXNIP) in at least one other experiment (Exp3) that used a completely different pair of T21 and D21 iPSC clones, which were grown by different experimentators at different time points and in different labs/countries (United Kingdom and Singapore). While the elevated Aβ aggregate levels in the T21 organoids compared to D21 was observed in both additional repeat experiments, showing the robustness of the role of chr21 triplication on Aβ accumulation, the difference was reproduced as significant only in Exp3. Similarly, inflammatory markers were also increased in both repeat experiments, but the difference was greater in Exp3. On the other hand there was no difference between TXNIP or AT8-positive tau levels between the D21 and T21 organoids in Exp2, that used the same disomic iPSC clone, but a different trisomic clone as compared to Exp3. Taken together, these results demonstrate the robustness of the system, reproducibility between individual iPSC clones, but also shows variations in the rates of pathogenesis between individual experiments. This has also been observed in monozygotic twins with familial AD (FAD) [104], while both twins developed AD, there were differences in the age of onset within the pairs. Similarly, changes in the rate of the severity of cognitive deficits has also been reported in FAD cases [105]. Our results, therefore show that it is possible to follow the disease progression by monitoring changes in the media of DS organoid samples and that understanding the basis of this variation might be important to understand why some people develop AD faster than others. Moreover, these results are also suggesting that, by following the temporal progression of individual pathologies in organoids developing at different rates, it may be possible to investigate the order in which changes occur. For example, while all of the markers (soluble Aβ and pTau aggregates, ASC, and TXNIP) were significantly higher in the T21 organoids studied at the first part of this study and in Exp3, only Aβ aggregates showed a significant difference in Exp2, where ASC showed a non-significant trend, and AT8-positive tau aggregates showed no difference. This shows a clear correlation between increased soluble pTau aggregates and markers of inflammasomes and oxidative stress, allowing us to speculate that early inflammation caused by exposure to toxic soluble Aβ aggregates might promote pTau aggregation in this model and there is variation between individual experiments as to the speed with which this Aβ-induced inflammation occurs. The variability of AD pathogenesis rates between different experiments therefore appears helpful to understand the order of the molecular processes giving rise to pathogenesis and more work needs to be done to understand the factors that lead to this observed variation in rate of AD pathogenesis, as now there is an established methodology to study these changes in organoids.

### Conclusions, limitations and future directions

In the first part of this study, we found increased soluble Aβ and pTau aggregates released by T21 organoids, suggesting increased Aβ may drive tau pathology in this model of AD. We did not observe time-related changes within the time window of culture (up to 150 DIV), suggesting a dynamic equilibrium is reached. Importantly, our study provides a proof of principle that soluble aggregates of both Aβ and tau show increased number and altered characteristics, accompanied by inflammasome formation and increased oxidative stress, preceding the development of plaque and tangle-like structures. Since we observe Aβ aggregates secreted also by the control organoids, our results suggest that aggregate-driven neuronal toxicity and plaque formation occur due to an imbalance between the rate of production and removal of aggregates. These results are in favour of the amyloid cascade hypothesis as the triplication of chr21, which contains the *APP* gene led to an accumulation of Aβ and importantly, pathological tau aggregation, along with increased inflammation and oxidative stress. These results establish a proof of principle that, cerebral organoids could enable future studies to test hypotheses about the mechanisms and sequence of early events of AD pathogenesis, and try therapeutics on a human-derived system. This has been a limitation on human samples as most sample collection happens post-mortem, making it highly difficult to perform controlled studies allowing interventions. Moreover, SiMPull and SiMoA were able to detect smaller, soluble aggregates significantly before the formation of plaques and tangle-like pathology, allowing us to study early pathological mechanisms, showing the importance of single-molecule techniques.

The second part of this study showed that despite the variability in the rate of pathogenesis between organoids grown in different experiments, the changes in markers of several pathogenic process components are conserved, making this model a powerful tool to study various aspects of AD. Nevertheless, this variability should be taken into account when using this model and the possible cause of this variability should be further studied. Moreover, the Aβ and AT8-positive aggregates, as well as ASC-specks and TXNIP were quantified and characterised in the conditioned media samples, however the intracellular disease mechanisms and the relationship between these disease markers have not been investigated. Even though AT8-positive tau has been associated with AD pathology and tau aggregation, and tau aggregates were specifically studied here (rather than monomeric tau), using further phosphorylated tau antibodies to study hyperphosphrylation would be beneficial to understand the extent of tau pathology in this model. This study shows the feasibility to characterise the AD-relevant soluble oligomeric aggregates in conditioned media samples from T21 organoids using super-resolution microscopy and on a broader scale studying pathological aggregates in conditioned media samples, opening up more detailed studies of the aggregation of soluble Aβ and tau in a realistic human model of disease in future work.

### Supplementary information

Supplemental Figure 1

Supplemental Figure 2

Supplemental Figure 3

Supplemental Figure 4

Supplemental figure legends

## Data Availability

The datasets generated and analysed during the current study are available from the corresponding author upon reasonable request.
