# Supplementary figures and images for: Cerebral organoids with chromosome 21 trisomy secrete Alzheimer’s disease-related soluble aggregates detectable by single-molecule-fluorescence and super-resolution microscopy
Source: Mol Psychiatry. 2023 Dec 15;29(2):369–86. doi: 10.1038/s41380-023-02333-3 (PMC11116105; doi:10.1038/s41380-023-02333-3)

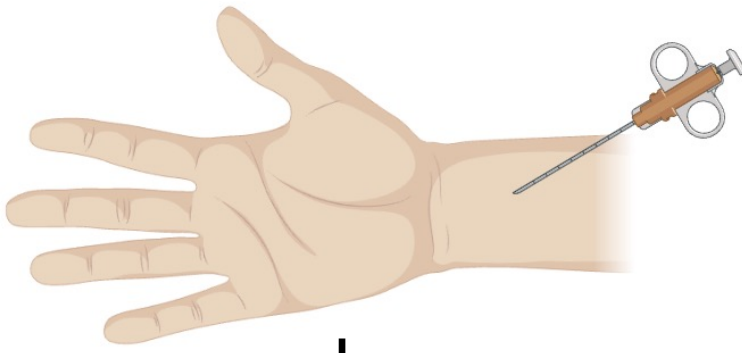

Skin biopsy from from mosaic  
DS individual: cytogenetics  
finds 60% T21, 40% D21

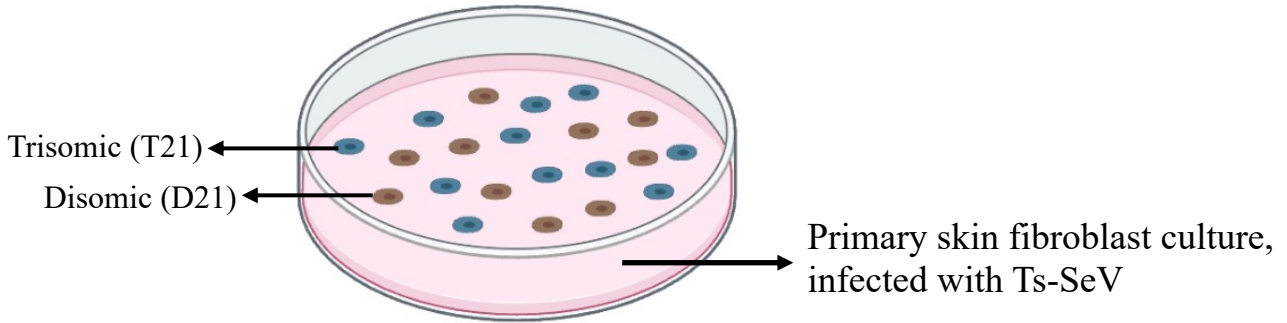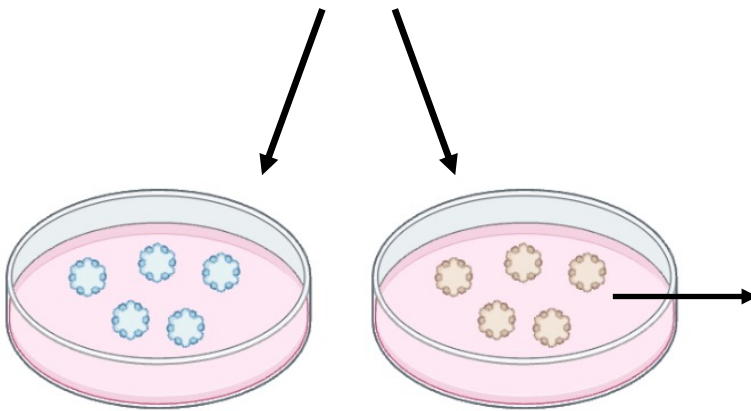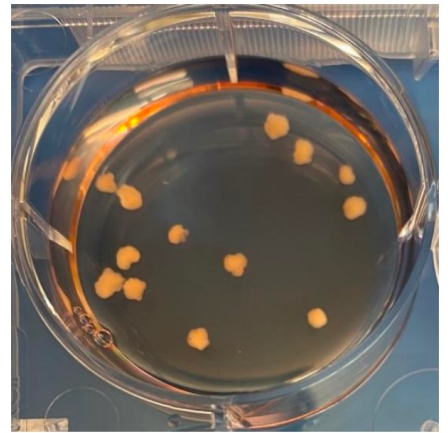

Supplement: Supplementary file 1 — Supplemental Figure 1 [file 41380_2023_2333_MOESM1_ESM.pdf]

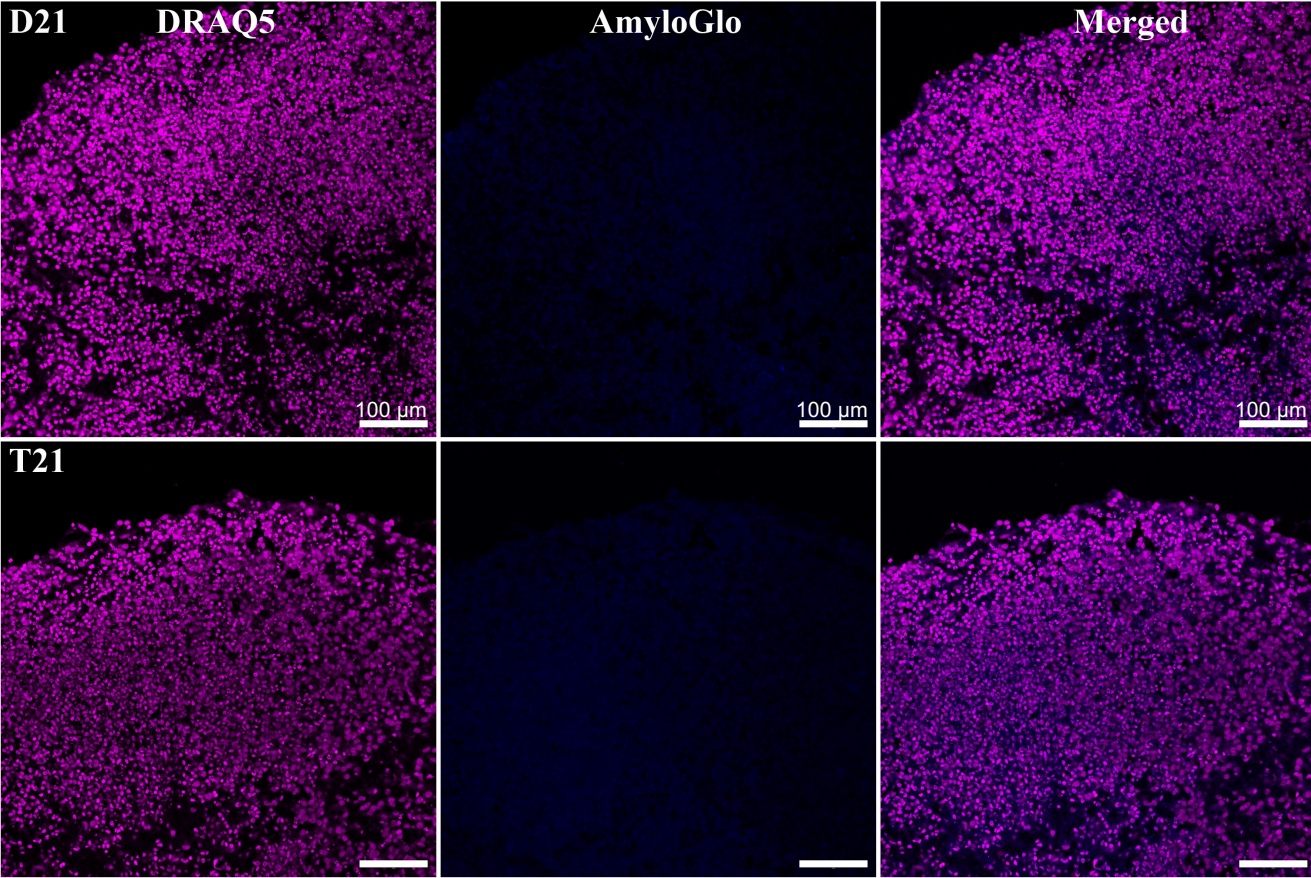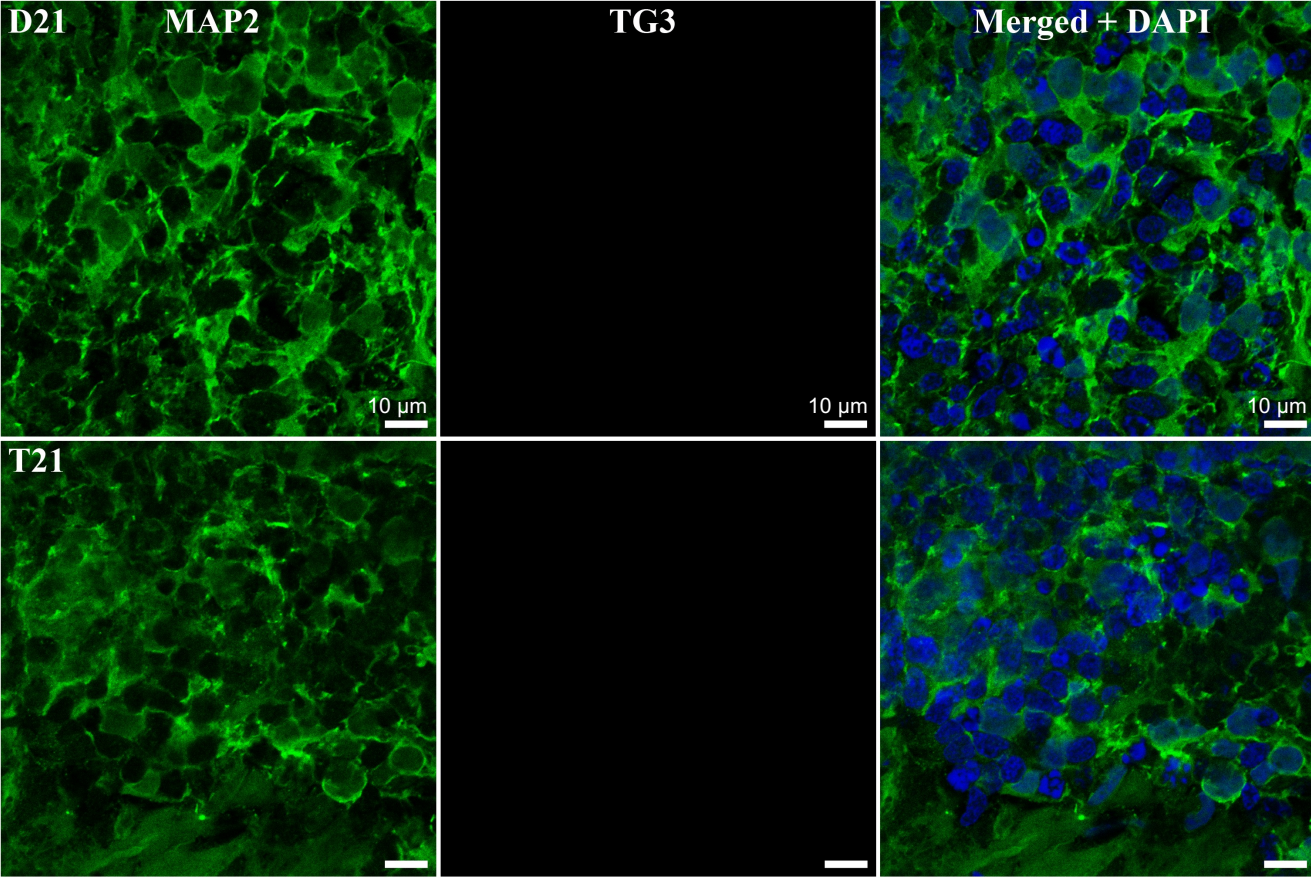

Supplement: Supplementary file 2 — Supplemental Figure 2 [file 41380_2023_2333_MOESM2_ESM.pdf]

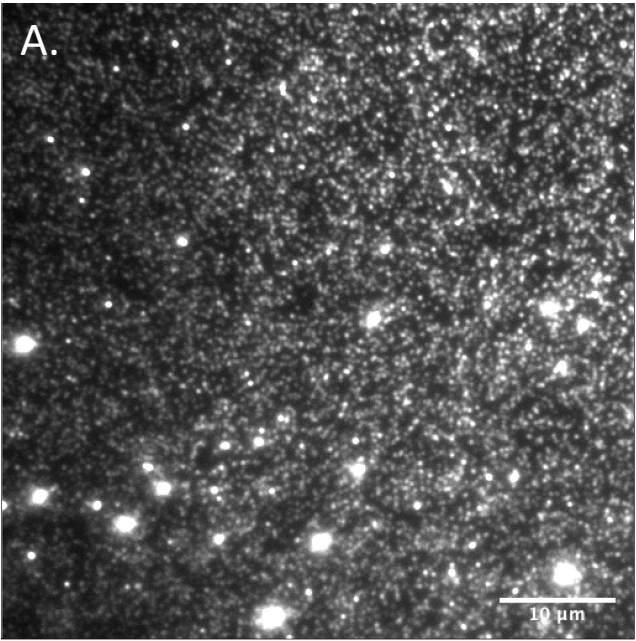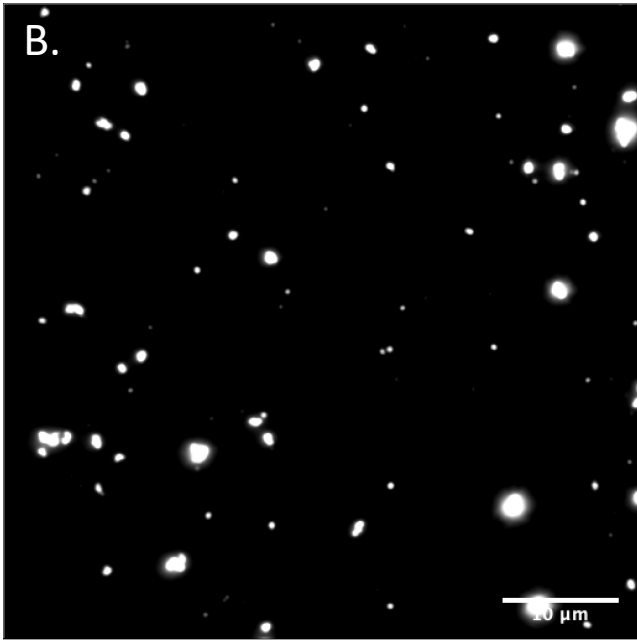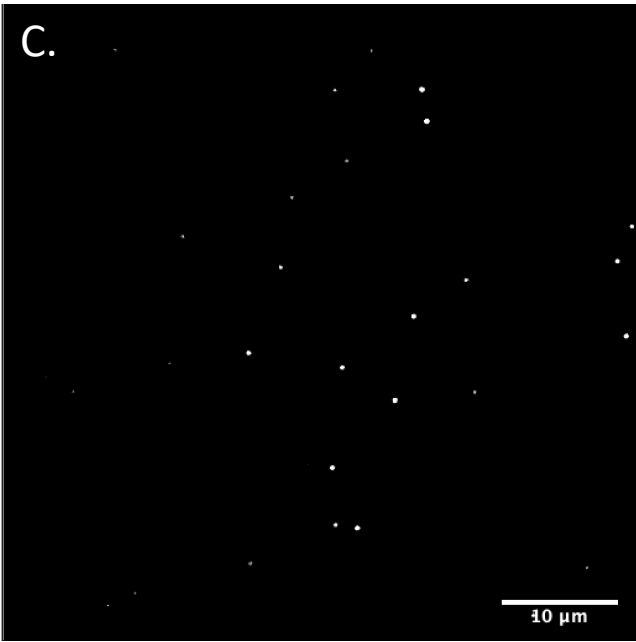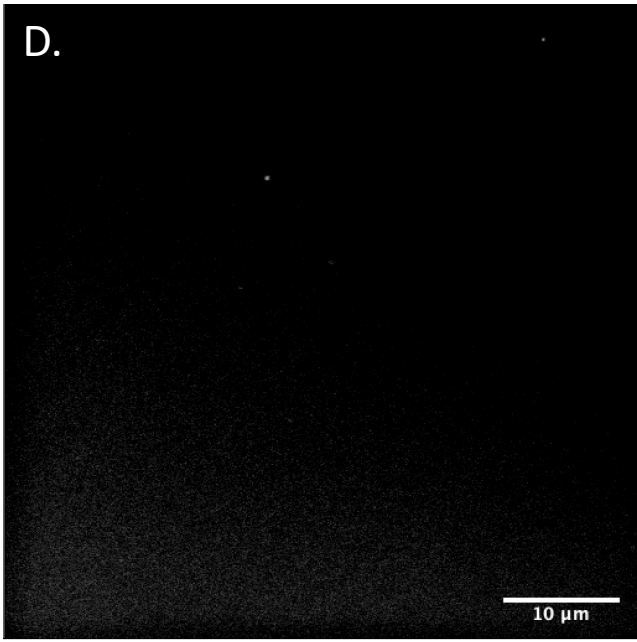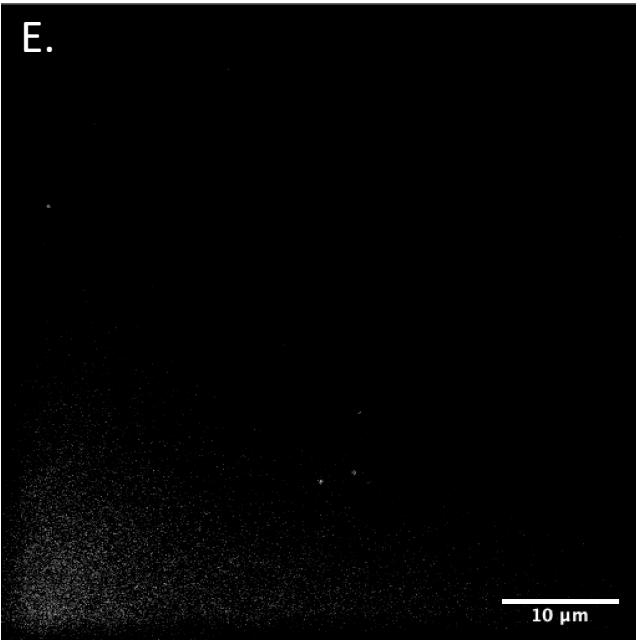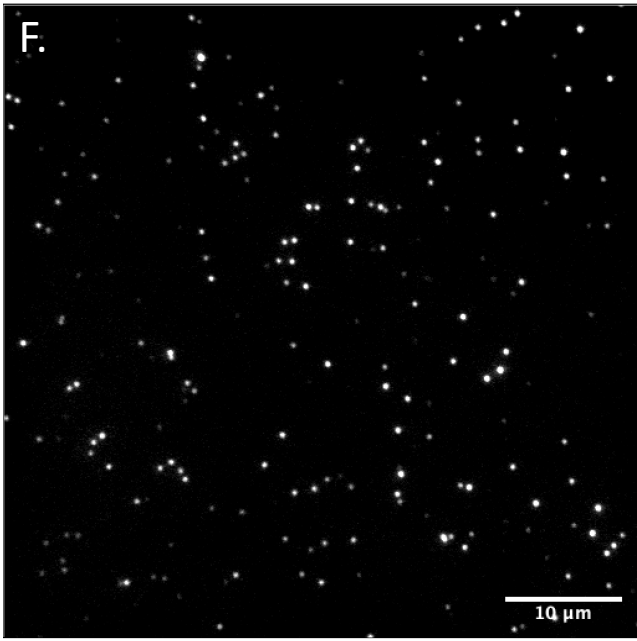

Supplement: Supplementary file 3 — Supplemental Figure 3 [file 41380_2023_2333_MOESM3_ESM.pdf]

A.

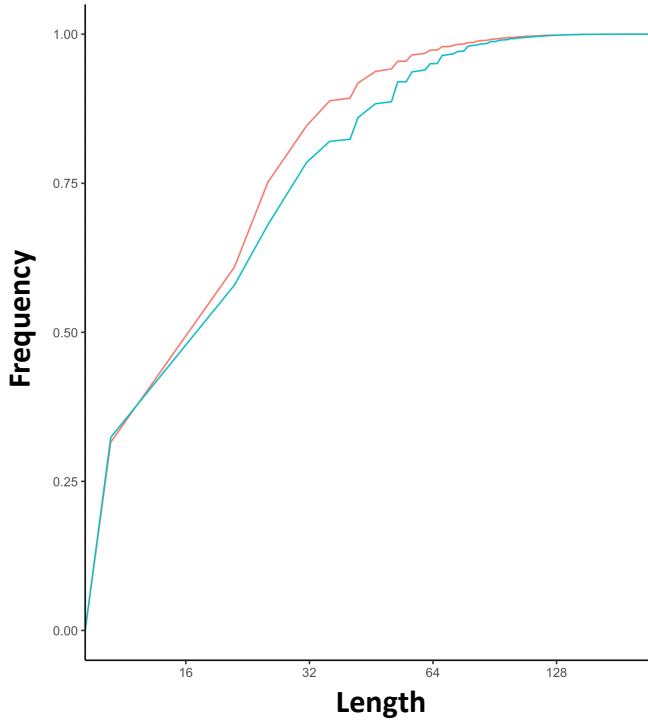

B.

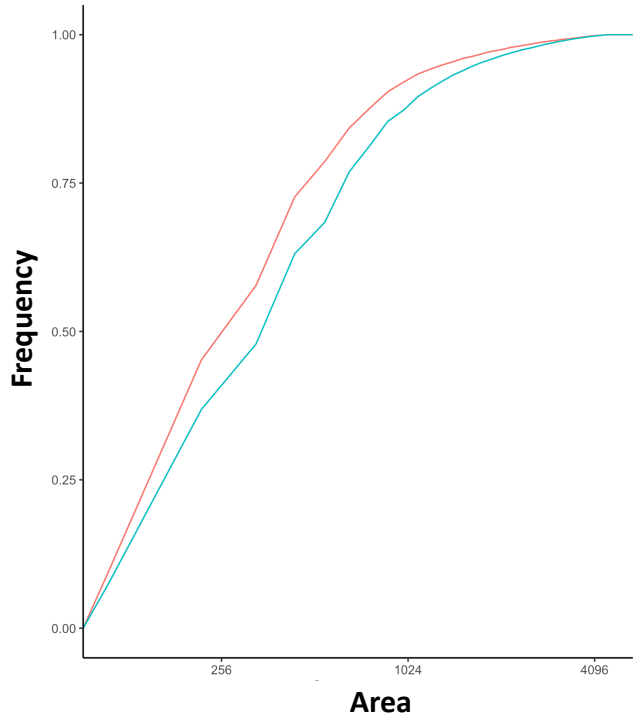

C.

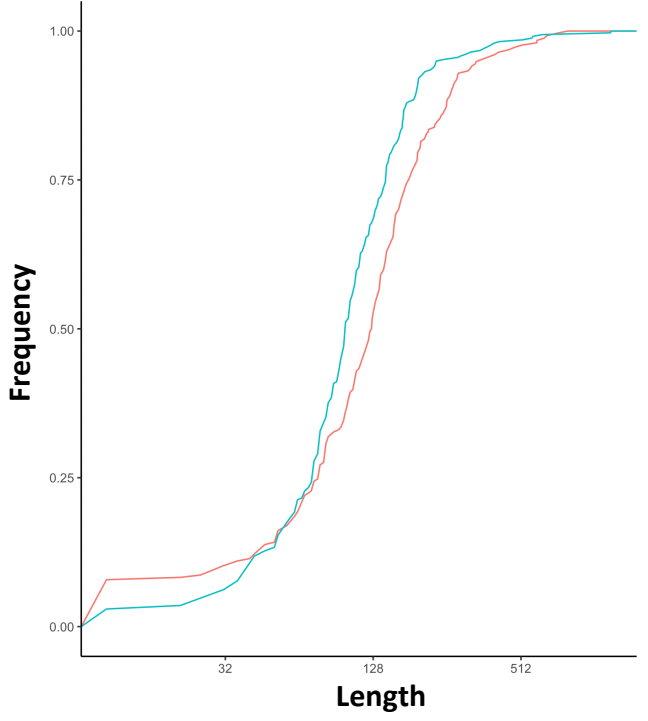

D.

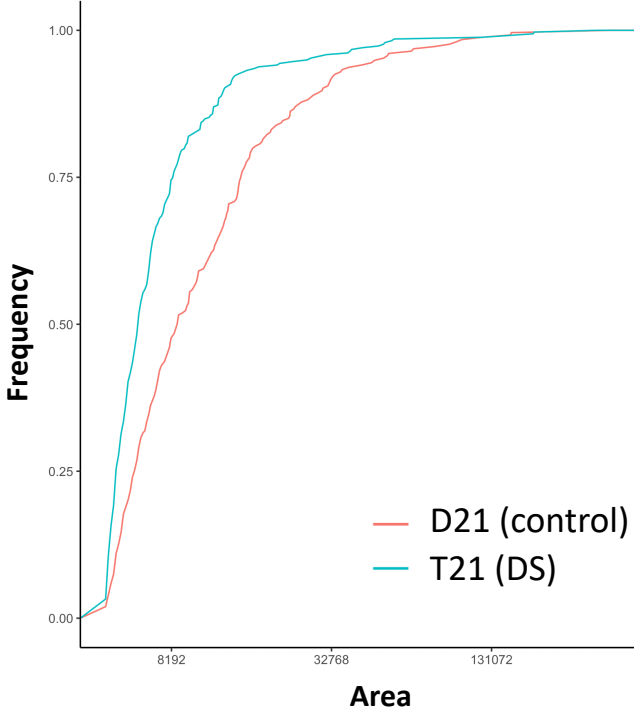

Supplement: Supplementary file 4 — Supplemental Figure 4 [file 41380_2023_2333_MOESM4_ESM.pdf]
