# Supplementary material for: Cerebral organoids with chromosome 21 trisomy secrete Alzheimer’s disease-related soluble aggregates detectable by single-molecule-fluorescence and super-resolution microscopy
Source: Mol Psychiatry. 2023 Dec 15;29(2):369–86. doi: 10.1038/s41380-023-02333-3 (PMC11116105; doi:10.1038/s41380-023-02333-3)
Supplement: Supplementary file 5 — Supplemental figure legends [file 41380_2023_2333_MOESM5_ESM.docx]

**Supplemental Figure 1.** Schematic representation of the culturing of D21 and T21 cerebral organoids. iPSC’s were generated from tissue collected during skin biopsy from an individual with mosaic Down’s syndrome.

**Supplemental Figure 2.** No beta-amyloid plaque-like (AmyloGlo) or tau tangle-like (TG3) histopathology was observed in the organoids used in this study. Nuclei are labelled with DRAQ5 or DAPI, and neurons are labelled with MAP2. Representative images from DIV100 organoids (Exp3) are shown. Scalebar is 100 μm for AmyloGlo images and 10 μm for TG3 images.

**Supplemental Figure 3.** Controls for beta-amyloid (Aβ) SiMPull. **A.** 1 uM synthetic Aβ42 captured with 6E10 and imaged with 12F4, where total Aβ (monomers and aggregates) can be seen or **B.** same preparation as in “A” but with the same capture and imaging antibody (6E10), whereby monomers are not imaged. **C.** 0.2 mg/ml BSA captured and imaged with 6E10 (blank control). **D.** Unconditioned media captured and imaged with 6E10 (control for media fluorescence). **E.** Randomly chosen sample D21 DIV105 captured with 6E10 and imaged with mouse IgG conjugated with AF647 (IgG control). **F.** Randomly chosen sample D21 DIV105 captured and imaged with 6E10 (positive control).

**Supplemental Figure 4.** Further characterisation of Aβ aggregates in the conditioned media samples by grouping them based on their area (smaller or larger than 4000 nm^2^). The cumulative predicted **A.** length and **B.** area distribution of the aggregates smaller than 4000 nm^2^ (98.5% of all aggregates). The cumulative predicted **C.** length and **D.** area distribution of the aggregates larger than 4000 nm^2^ (1.5% of all aggregates).
